# Nuclear poly(A)-binding protein 1 is an ATM target and essential for DNA double-strand break repair

**DOI:** 10.1093/nar/gkx1240

**Published:** 2017-12-14

**Authors:** Michal Gavish-Izakson, Bhagya Bhavana Velpula, Ran Elkon, Rosario Prados-Carvajal, Georgina D Barnabas, Alejandro Pineiro Ugalde, Reuven Agami, Tamar Geiger, Pablo Huertas, Yael Ziv, Yosef Shiloh

**Affiliations:** 1 Department of Human Molecular Genetics and Biochemistry, Sackler School of Medicine, Tel Aviv University, Tel Aviv, Israel; 2 Centro Andaluz de Biología Molecular y Medicina Regenerativa (CABIMER) and Department of Genetics, University of Sevilla, Sevilla, Spain; 3 Division of Biological Stress Response, The Netherlands Cancer Institute, Amsterdam, The Netherlands

## Abstract

The DNA damage response (DDR) is an extensive signaling network that is robustly mobilized by DNA double-strand breaks (DSBs). The primary transducer of the DSB response is the protein kinase, ataxia-telangiectasia, mutated (ATM). Here, we establish nuclear poly(A)-binding protein 1 (PABPN1) as a novel target of ATM and a crucial player in the DSB response. PABPN1 usually functions in regulation of RNA processing and stability. We establish that PABPN1 is recruited to the DDR as a critical regulator of DSB repair. A portion of PABPN1 relocalizes to DSB sites and is phosphorylated on Ser95 in an ATM-dependent manner. PABPN1 depletion sensitizes cells to DSB-inducing agents and prolongs the DSB-induced G2/M cell-cycle arrest, and DSB repair is hampered by PABPN1 depletion or elimination of its phosphorylation site. PABPN1 is required for optimal DSB repair via both nonhomologous end-joining (NHEJ) and homologous recombination repair (HRR), and specifically is essential for efficient DNA-end resection, an initial, key step in HRR. Using mass spectrometry analysis, we capture DNA damage-induced interactions of phospho-PABPN1, including well-established DDR players as well as other RNA metabolizing proteins. Our results uncover a novel ATM-dependent axis in the rapidly growing interface between RNA metabolism and the DDR.

## INTRODUCTION

The double-strand break (DSB) is a severe DNA lesion when generated by internal or external DNA damaging agents. Failure to repair DSBs has major consequences for genome integrity and cell fate, and may result in undue cell death or genomic rearrangements that may lead to cancer formation (1,2). DSBs vigorously trigger the DNA damage response (DDR), an elaborate signaling network that reaches out to all cellular compartments and mobilizes numerous cellular processes (3–5). This network is based on a core of dedicated DDR players and vast, temporary recruitment of additional proteins from other physiological circuits. DSB repair is conducted by a highly coordinated spatiotemporal cascade that begins with massive recruitment of DSB ‘sensors’ to DNA breaks (6), and subsequent transmission of a signal to protein kinases that act as transducers that relay the signal to numerous downstream effectors. Two major DSB repair pathways are utilized: end-resection-independent, canonical nonhomologous end-joining (C-NHEJ) and resection-dependent homologous recombination repair (HRR) (5,7). Additional, minor resection-dependent pathways are single-strand annealing (SSA) and alternative end-joining (Alt-EJ) reviewed in (7,8). Of these pathways, only HRR is error-free. In higher eukaryotes, the predominant DSB repair pathway throughout the cell cycle is C-NHEJ, which rejoins broken ends after their processing (9). The HRR pathway, which is active only in the late S and G2 phases of the cell cycle, is based on homologous recombination using the intact sister chromatid as a template to accurately retrieve the missing information in the broken copy, making it error-free (8,10). A delicate balance exists between the different repair pathways, which is influenced by cell type, cell cycle stage and the structure and amount of DSBs. Interference with this balance may abrogate DSB sealing or increase the extent of error-prone repair, elevating genomic aberrations (11–13).

The assembly of the cellular response to DSB is based on a wide range of protein posttranslational modifications (PTMs) (14–16). The predominant damage-induced PTMs are poly(ADP-ribosylation), phosphorylation and modification by the ubiquitin family proteins. Phosphorylation typically marks many proteins that are recruited to DNA damage sites as well as core histones in the vicinity of DNA breaks. The chief transducer of this massive response is the serine–threonine protein kinase, ataxia-telangiectasia mutated (ATM), which is activated following DSB induction and in turn phosphorylates a plethora of effectors in various DDR pathways (17–19). ATM is a homeostatic protein kinase with functions in many cellular circuits (18,20). It is a member of the PI3 kinase-related protein kinase (PIKK) family, which includes, among others, the catalytic subunit of the DNA-dependent protein kinase (DNA-PKcs) (21,22) and the A-T and RAD3-related protein (ATR) (23). The three protein kinases maintain a complex functional crosstalk in response to various genotoxic stresses (19,24–26). Key ATM effectors modulate biological pathways that affect numerous physiological circuits. Thus, the investigation of new branches of this network often leads to different aspects of cellular physiology.

The wealth of potential DDR players borrowed from the RNA metabolism, which were detected in many screens for new DDR players (27–31), points at a growing, broad interface between the DDR and the RNA arenas. Indeed, besides global approaches, work focusing on specific RNA binding proteins (RBPs) has highlighted their roles in the DDR (32–38). They regulate the levels of DDR proteins at various post-transcriptional levels, regulate R-loop formation and formation of hazardous DNA topology at damage sites, and play direct roles in DNA repair. Yet our knowledge of this increasingly appreciated link between the DDR and RNA metabolism is limited, especially when it comes to focused studies on individual players and understanding their functional significance and the relevant mechanisms. We came across a novel player in this intriguing coalesce when nuclear poly(A)-binding protein 1 (PABPN1) was identified as potential ATM substrate in a phosphoproteomic screen carried out in our laboratory in order to explore the DSB-induced dynamics of the nuclear phosphoproteome (31).

PABPN1 plays an important role in various aspects of RNA processing and stability (39): it binds poly(A) tails of pre-mRNAs while stimulating polyadenylation (40–42), and was recently shown to be a suppressor of alternative cleavage and polyadenylation (APA) (43,44). APA is a widespread post-transcriptional phenomenon that contributes to the complexity of the transcriptome by generating isoforms that differ either in their coding sequence or in their 3′ untranslated regions (UTRs) (45). Here, we show that PABPN1 is an ATM substrate, which plays a role in regulating DSB repair. Proteomic analysis suggests that in response to DNA damage PABPN1 interacts with several known and new DDR players including other regulators of mRNA metabolism.

## MATERIALS AND METHODS

### Chemical reagents, antibodies and immunoblotting

DharmaFECT1 transfection reagent was obtained from Dharmacon (GE Dharmacon, Lafayette, CO, USA). Neocarzinostatin (NCS) was obtained from Sigma–Aldrich (St. Louis, MO, USA). The ATM inhibitor, KU55933 (46), the DNA-PK inhibitor, NU7441 (47) and the ATR inhibitor, AZ20 (48) were obtained from Tcris Bioscience (Bristol, UK). Anti-53BP1 mouse monoclonal antibody was a generous gift from T. Halazonetis, anti-53BP1 polyclonal antibody was obtained from Novus Biologicals, LLC (Littleton CO, USA), anti-pS139-H2AX monoclonal antibody was obtained from Merck Millipore (Darmstadt, Germany), anti-PABPN1 and anti-BRCA1 antibodies were obtained from Abcam (Cambridge, MA, USA), phospho-antibodies against phosphorylated Ser 95 of PABPN1 and phosphorylated KAP-1 were obtained from Bethyl Laboratories, Inc. (Montgomery, TX, USA), anti-ATM obtained from Sigma–Aldrich (St. Louis, MO, USA), anti-HSC70 monoclonal antibody was obtained from Santa Cruz Biotechnology Inc. (Santa Cruz, CA, USA), anti-RAD51 was obtained from Chalbiochem, Inc. (San Diego, CA, USA), antimouse and anti-rabbit IgG, Alexa 488/568/633 were purchased from Molecular Probes (Leiden, Netherlands), and HRP-conjugated anti-rabbit IgG and anti-mouse IgG were obtained from Jackson Immunoresearch Laboratories, Inc. (West Grove, PA, USA). Immunoblotting was carried out as previously described (49).

### Cell lines

HeLa and U2-OS cells were grown in DMEM medium with 10% fetal bovine serum at 37°C in 5% CO_2_ atmosphere.

### Vectors and constructs

PABPN1 cDNA cloned in pCMV-AC-GFP and PABPN1 cDNA resistant to siRNA2—WT or mutated in serine95 (S95A, S95D)—cloned in pcDNA5/FRT/V5-His TOPO vector, were a kind gift from R. Agami (Netherlands Cancer Institute, Amsterdam, The Netherlands). PABPN1 cDNA resistant to siRNA2—WT or mutated on serine95 (S95A, S95D)—was cloned in pEGFP-C1 vector.

### RNA interference

siRNAs were obtained from Dharmacon. Two custom designed siRNAs were used to target PABPN1 (siRNA1: UUAGAUGAGUCCCUAUUUA, siRNA2: AGUCAACCGUGUUACCAUAUU). siRNA targeting ATM (GACUUUGGCUGUCAACUUUCG), irrelevant siRNAs targeting Luciferase (CGUACGCGGAAUACUUCGA) or GFP (GGAGCGCACCATCTTCTTC) carry the OnTarget Plus modifications. Cells were grown to 50% confluence and transfected with siRNA using DharmaFECT1 transfection reagent according to manufacturer's instructions.

### Immunoprecipitation

U2-OS cells were harvested and lysed in RIPA lysis buffer or in 0.5% NP40 for CO-IP. 0.5–1 mg protein extract was incubated with an antibody against PABPN1 or with IgG (control) and incubated overnight, rotating, at 4°C. The rest of the extract was collected and kept as an input. Protein-A sepharose beads were washed four times and centrifuged between each time (5000 RPM, 2 min, 4°C) and incubated with the immune complexes for 1 h while rotating at 4°C. Immune complexes were pulled-down (5000 RPM, 2 min, 4°C). Supernatants were collected and the pellets were washed four times and alternately centrifuged (5000 RPM, 2 min, 4°C). Total cell extract, immune complexes, and supernatants were boiled in sample buffer, loaded on 8–10% SDS-PAGE and transferred onto a nitrocellulose membrane.

### Colony forming assay

Hela cells double-transfected with siRNA against ATM, PABPN1 and non-targeted control siRNA were plated in triplicates at densities of 100–5000 cells per 60 mm plate. Seventy two hours after transfection, cells were treated with various doses of NCS (5, 7.5, 10, 12.5 ng/ml) or IR (2, 4, 8, 12 Gy). Cells were grown for 10–14 days and then were fixed and stained with 2% crystal violet in 50% ethanol. Colonies were counted using a dissection microscope.

### Cell-cycle analysis

U2-OS cells were transfected with siRNA against ATM, PABPN1 and Luciferase. Seventy two hours after transfection, cells were treated with 3–4 ng/ml NCS and harvested at various time points. Cell-cycle checkpoint analysis was carried out using a BD ACCURI C6 flow cytometer (Ann Arbor, MI, USA) after DNA staining with propidium iodine. Statistical analysis of results was carried out using the FlowJo software (Ashland, OR, USA).

### Laser-induced localized DSBs

U2-OS cells were grown on glass-bottomed 35 mm dishes (Matek Corp., Ashland, MA, USA) and transfected with GFP-tagged PABPN1 using Fugene6 (Roche). Twenty four hours later cells were irradiated with a focused 800 nm laser beam in a Zeiss LSM 510 Meta confocal microscope equipped with a Spectra-Physics Mai-Tai multiphoton laser; GFP-tagged PABPN1 was monitored.

### Immunofluorescence

U2-OS cells were plated on 22 mm glass coverslips, treated with 3.5 ng/ml NCS or 1 Gy of IR, fixed in 4% buffered paraformaldehyde for 15 min, and permeabilized in PBS containing 0.5% Triton X-100. Coverslips were blocked for 15 min with 10% bovine serum albumin in PBS, incubated after NCS treatments for 1.5 h at room temperature with primary antibodies diluted in primary antibody dilution buffer (Biomeda Corp., Foster City, CA, USA), and washed and incubated with secondary antibody for 30 min. Cell nuclei were stained with DAPI. The coverslips were washed three times with PBS after each step. The procedure was performed at room temperature. Nuclear foci were quantified using the ImageJ software (http://imagej.nih.gov/ij/). 200–300 cells were counted per time point.

### DSB repair pathway assays

U2-OS cells bearing a single copy integration of the reporters DR-GFP (Gene conversion (50), EJ2 (AltNHEJ) and EJ5 (NHEJ) (51) or SSR (NHEJ/Recombination balance (52)) were used to analyze the different DSB repair pathways. In all cases, cells were plated in 10 cm dishes. One day after seeding, they were infected with a lentivirus harboring an I-SceI and labeled with BFP (53) using a multiplicity of infection (M.O.I) of 5. Six hours after infection the same volume of fresh medium was added. Cells were grown for 48 h, fixed with 4% paraformaldehyde, and washed with PBS prior to visualization with a fluorescent microscope for blue, green and, in the case of the SSR, red fluorescence. The repair frequency was calculated as the percentage of blue cells expressing GFP for the DR-GFP (gene conversion), EJ5 (NHEJ) and EJ2 (MMEJ) reporters. For the HR/NHEJ balance, the ratio of green to red cells in each condition was calculated as published (52). To facilitate the comparison between experiments, this ratio was normalized with a control transfected with a control siRNA. For the SSR, conditions that skew the balance towards an increase in NHEJ repair result in a fold increase <1, whereas an imbalance of the SSR towards HRR results in a fold increase >1. Data represent a minimum of three sets of duplicated experiments.

### DNA end-resection

SMART (Single Molecule Analysis of Resection Tracks) was performed as previously described (54). Briefly, U2-OS cells transfected with either siRNA against PABPN1 or a control siRNA were cultured in the presence of 10 M bromodeoxyuridine (BrdU) for 24 h, irradiated with 10 Gy of IR, and harvested 1 h later. Cells were embedded in low-melting agarose, and in-gel DNA extraction followed. To stretch the DNA fibers, silanized coverslips (Genomic Vision, Bagneux, France) were dipped into the DNA solution for 15 min and pulled out at constant speed (250 m/s). Coverslips were baked for 2 h at 60°C and incubated without denaturation with an anti-BrdU mouse monoclonal antibody. After washing with PBS, coverslips were incubated with a secondary antibody for 1 h at room temperature. The coverslips were mounted with ProLong R_Gold AntifadeReagent (Molecular Probes) and stored at −20°C. DNA fibers were observed with a Nikon NI-E microscope under a PLAN FLUOR DLL 40×/0.75 PHL objective. The images were recorded and processed using NIS ELEMENTS Nikon software. For each experiment, at least 200 DNA fibers were analyzed, and the length of DNA fibers was measured using Adobe Photoshop CS4 Extended version 11.0 (Adobe Systems Incorporated). Statistical significance in these experiments was determined with the paired Student's t-test using the PRISM software (Graphpad Software Inc.).

### Immunoprecipitation mass spectrometry

U2-OS cells were transfected with siLuciferase or siPABPN1 (as control), treated 72 h later with 50 ng/ml NCS for 30 min, harvested and kept at –80°C. Cell pellets were thawed on ice, resuspended in 1 ml lysis buffer (1% IGEPAL-CA- 630 (NP-40), 1 mM MgCl2, 1× protease inhibitors, EDTA-free, 1% Benzonase, 150 mM NaCl 50 mM, Tris–HCl (pH 7.5)) for 30 min at room temperature and centrifuged at 13 000 × g at 4°C for 15min. Supernatants were transferred to new tubes and incubated with pPABPN1 or total PABPN1 antibodies overnight at 4°C while rotating. Protein-A sepharose beads were washed four times and centrifuged between each time (5000 RPM, 2 min, 4°C) and incubated with the immune complexes for 1 h while rotating at 4°C. The immunoprecipitated complexes were washed three times with buffer I (0.05% IGEPAL-CA-630 (NP-40), 150 mM NaCl, 50 mM Tris–HCl (pH 7.5)) followed by three times with buffer II (150 mM NaCl, 50 mM Tris–HCl (pH 7.5)). The complexes were trypsin-digested and eluted with 100 μl elution buffer I (2 M urea, 50 mM Tris–HCl -pH 7.5, 1 mM DTT and 0.4 μg sequencing grade trypsin) at room temperature for 2 h. The supernatants were transferred to new tubes and eluted with 100 μl elution buffer II (2 M urea, 50 mM Tris–HCl pH 7.5 and 5 mM iodoacetamide). The proteins were digested overnight, and the digestion was terminated by 0.1% trifluoroacetic acid (TFA). The peptides were desalted, concentrated on C18 stage tips, eluted using 80% acetonitrile, vacuum-concentrated and diluted in loading buffer (2% acetonitrile and 0.1% trifluoroacetic acid). The eluted peptides were loaded onto a 50 cm long EASY-spray reverse phase column coupled on line to a Q Exactive Plus mass spectrometer. Peptides were separated using a 240-min linear gradient of water and acetonitrile. Raw files were analyzed with MaxQuant (55) software (version 1.5.2.10), with an - FDR threshold of 0.01 on the peptide and protein identification. Bioinformatics and statistical analyses were performed using the Perseus program (56). To identify the proteins that interact specifically with p-PABPN1, log2 protein intensities of siLuciferase were compared to siPABPN1 samples and proteins >2-fold expression in at least two of three replicates were selected as the interactors. Similar analysis were done for total PABPN1 interactors. Protein-protein interaction network for phospho and total PABPN1 were performed in Cytoscape (3.2.1).

### Deposition of mass-spectrometric data

The data are available via ProteomeXchange with identifier PXD005913.

### 3′-Seq

The main 3′-Seq experiment (using 20 ng/ml NCS) constructed sequencing libraries using the protocol described in [PMID: 22747694]. The follow-up experiment using 5 or 10 ng/ml NCS applied a slightly modified protocol described in [PMID: 26671978].

### Analysis of 3′-Seq data

Sequenced reads were aligned to the human genome (hg19) using Bowtie [REF: PMID: 19261174]. Up to two mismatches were permitted in the reads' seed region (the reads' first 28 nucleotides). To allow the alignment of reads that span poly(A) CSs, and therefore contain the start of the untemplated poly-A tail, Bowtie's -e parameter was increased to tolerate mismatches in all bases after the seed region. Only uniquely mapped reads were used in subsequent analysis (Supplementary Table S1). To map reads to genes and genomic regions (for example, 3′ UTRs, introns, coding sequences, and so on), gene coordinates and annotations were extracted from the human Ensembl-Gene table of the University of California, Santa Cruz (UCSC) browser. Uniquely mapped reads that contained untemplated stretches of As were used to identify CSs (Supplementary Figure S4). To reduce false calls stemming from priming of the oligo-dT primer to internal A-rich regions within transcripts, we required that, to support a CS call, reads should contain a stretch of at least eight As and at least five of the first eight As in the stretch should mismatch the corresponding bases on the transcript reference sequence. The location of the cleavage was taken as the location where the untemplated A stretch started. Since the location of the CSs often fluctuated around a major site, we calculated for each gene and sample a ‘poly(A) CS profile’ that recorded the number of reads supporting a cleavage at each position along the transcript. We considered the local maxima of these CS profiles the CS locations, and required spacing of at least 50 nucleotides between consecutive CSs (in case of lower spacing between CSs, we chose the stronger, that is, the one supported by a higher number of reads). Only CSs supported by at least ten reads were considered in subsequent analyses. Differential usage of CSs was identified using chi-square test (and BH-FDR correction for multiple testing).

### RNA-seq

RNA-seq data from PABPN1-depleted and control cells were used to estimate changes in gene expression that result from attenuated expression of PABPN1. Expression levels were estimated by rpkm-normalized counts of the number of reads that map to each gene followed by quantile normalziation. Fold-change (log_2_) in expression were subsequently calculated per gene between the PABPN1-depleted and control samples. To avoid inflation of high fold-change levels for lowly expressed genes, expression levels <1.0 were set to 1.0. Sequenced reads were aligned to the human genome (hg19) using tophat. Only uniquley mapped reads were considered.

## RESULTS

### PABPN1 is phosphorylated in an ATM-dependent manner on Ser95 following DSB induction

**Figure 1.**
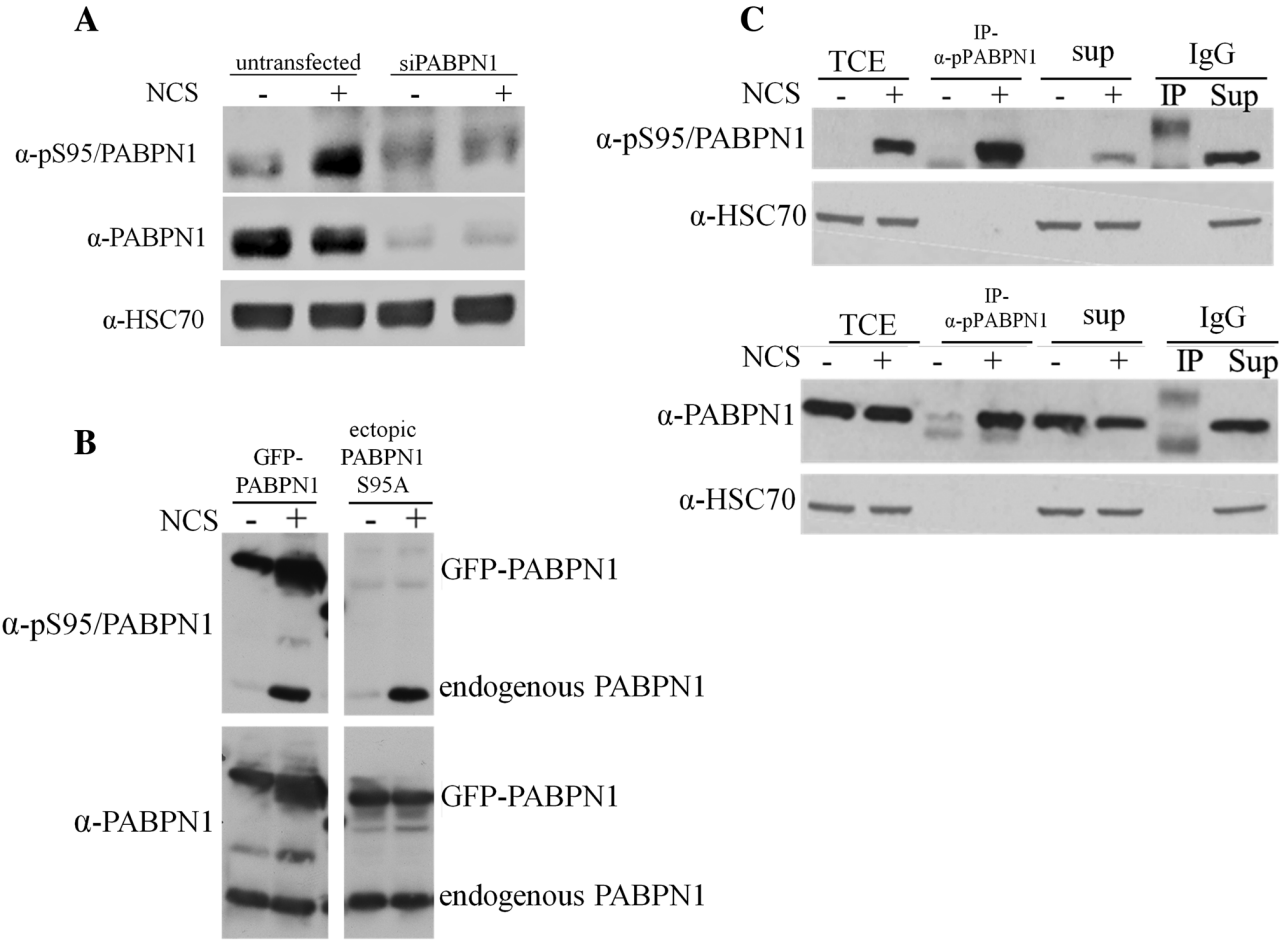
PABPN1 is phosphorylated on Ser95 in response to DSB-inducing agents. (**A**) Detection of phosphorylated PABPN1 in cellular extracts using a phospho-specific antibody raised against the presumed phosphorylation site of PABPN1 on Ser95. PABPN1-depleted or -proficient U2-OS cells were treated with 20 ng/ml of the radiomimetic chemical, NCS, for 1 hr and cellular extracts were subjected to immunoblotting analysis using the indicated antibodies. (**B**) Examination of antibody specificity and validation of Ser95 phosphorylation. U2-OS cells expressing ectopic GFP-PABPN1 in WT or mutant (S95A) versions were treated with 20 ng/ml of NCS for 30 min and cellular extracts were subjected to immunoblotting analysis with the indicated antibodies. Note the signal obtained using the phospho-specific antibody with the WT protein, but not the mutants. (**C**) Only a portion of cellular PABPN1 is phosphorylated in response to DNA damage. Anti-pS95/PABPN1 antibody was used for immunoprecipitation of pPABPN1 from extracts of U2-OS cells treated with 50 ng/ml NCS for 30 min, or untreated cells. Total cell extract, immune complexes and supernatants were subjected to immunoblotting analysis with the indicated antibodies. (TCE = total cell extract; SUP = supernatant; IgG- served as a control).

PABPN1 was identified in our lab as a target of ATM-dependent phosphorylation in a screen that monitored nuclear phosphoproteome dynamics in response to DNA damage (31). Furthermore, the phosphorylation site was identified in that screen on Ser95 of this protein. In order to validate this phosphorylation, a phospho-specific, polyclonal antibody was raised against the presumed phosphorylated form of PABPN1. Following 30 min of treatment with the DSB-inducing chemical, neocarzinostatin (NCS), we observed in cellular extracts a signal that was markedly reduced upon PABPN1 depletion using RNAi (Figure 1A). To verify the specificity of the antibody to pS95 of PABPN1, we expressed in cells ectopic, GFP-tagged PABPN1 in wild-type (WT) or in a S95A mutant non-phosphorylatable version. Indeed, the S95A substitution completely abolished PABPN1 phosphorylation (Figure 1B), substantiating Ser95 as the phosphorylation site and attesting to the antibody's specificity.

To assess the fraction of cellular PABPN1 that is phosphorylated in response to DNA damage, we used the phospho-specific antibody for immunoprecipitation from extracts of NCS-treated cells and subjected the immune complexes and supernatant to immunoblotting with antibodies against phosphorylated or total PABPN1. Only a portion of the cellular content of PABPN1 was found to be phosphorylated following NCS treatment (Figure 1C). This is typical of many DDR players that are borrowed from other arenas. Notably, we observed PABPN1 phosphorylation also in response to two other DSB inducers, ionizing radiation (IR) and etoposide (Supplementary Figure S1A and B).

**Figure 2.**
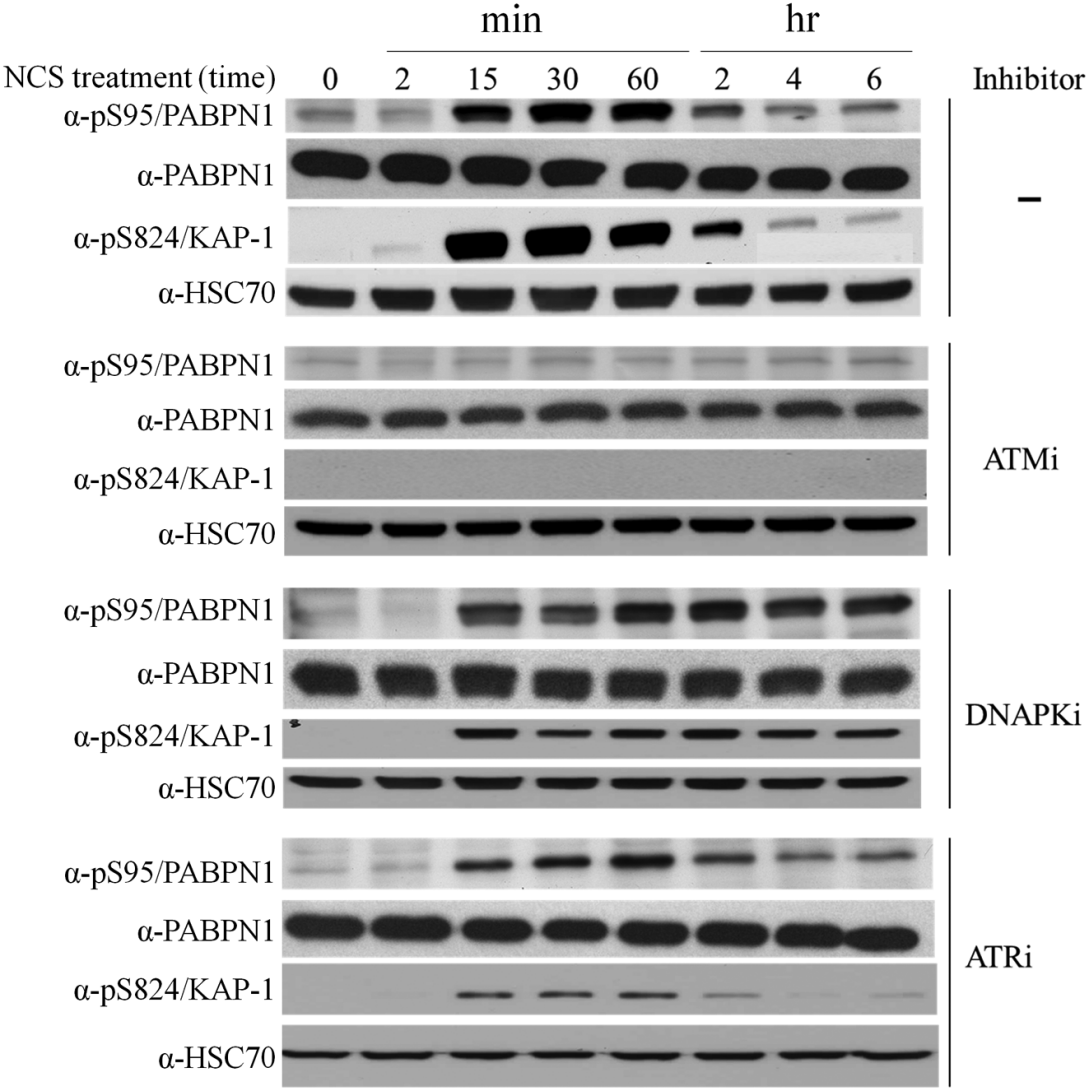
PABPN1 is a novel ATM substrate in the DSB response. ATM dependence of PABPN1 phosphorylation following NCS treatment. U2-OS cells were treated with 20 ng/ml of NCS, with or without 30 min pretreatment with 10 μM of the chemical ATM inhibitor, KU-55933. Cellular extracts were subjected to western blotting analysis with the indicated antibodies. Phosphorylated KAP-1 (57) served as control for DNA damage induction and response. PAPBN1 phosphorylation following NCS treatment is independent of ATR or DNA-PK. U2-OS cells were treated with 20 ng/ml of NCS, with or without pretreatment with the chemical inhibitors NU7441 (DNA-PK inhibitor-10 μM) or AZ20 (ATR inhibitor-0.5 μM). Cellular extracts were subjected to immunoblotting analysis with the indicated antibodies. Phosphorylated KAP-1 served as control for DNA damage induction and response.

The ATM dependence of PABPN1 phosphorylation, which had been reflected in the results of the screen that discovered it as potential DDR player (31), could now be readily validated using an ATM inhibitor (Figure 2). Notably, inhibitors of ATR or DNA-PK did not reduce this phosphorylation following NCS treatment (Figure 2). In cells treated with a DNA-PK inhibitor, no reduction in the damage-induced phosphorylation PABPN1 or KAP-1, a documented ATM substrate (57), was observed over time. Notably, it has been recently shown that DNA-PK inhibition leads not only to DSB persistence due to the hampered function of NHEJ but also to persistence of ATM activation due to loss of inhibitory effect of DNA-PK on ATM activity (58). The PIKKs ATM, DNA-PK and ATR target mainly S/TQ motifs (59,60). Indeed, Ser95 of PABPN1 is followed by a glutamine residue, making it a likely ATM target.

PABPN1 phosphorylation was characterized by rapid kinetics, which is typical of many ATM targets, peaking 30–60 min after DSB induction and largely decaying within 3 hours (Supplementary Figure S2A). Finally, PABPN1 phosphorylation intensified with increasing doses of NCS or IR (Supplementary Figures S2A, S1A). Collectively, these results establish Ser95 of PABPN1 as an ATM target in response to DSB induction and suggest a role for PABPN1 in the ATM-dependent DSB response.

### PABPN1-deficient cells present NCS hypersensitivity and prolonged cell-cycle arrest in response to DSB induction

**Figure 3.**
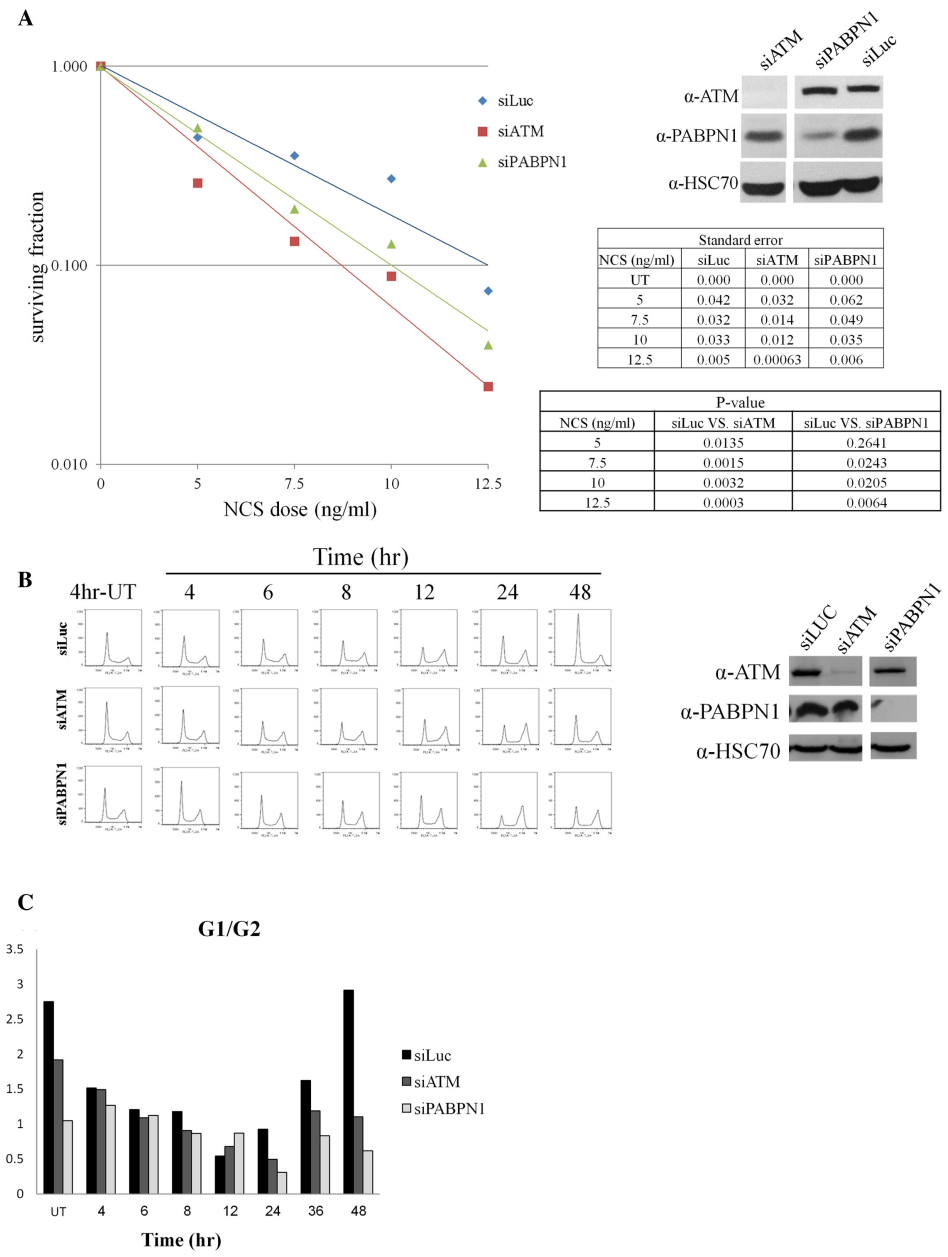
PABPN1 plays a role in the DDR. (**A**) PABPN1-depleted cells exhibit NCS hypersensitivity. Shown are **s**urvival curves based on clonogenic growth of HeLa cells transfected with siRNA against Luciferase (control), or ATM or PABPN1, and treated with increasing doses of the NCS. The data represent three sets of triplicate experiments. Statistical analysis was based on Student's t test. The tables present p-values and SEM. Immunoblotting analysis present the extent of protein depletion in this experiment. (**B**) U2-OS cells transfected with siRNAs against ATM, or PABPN1 or Luciferase were treated with 3.5 ng/ml of NCS and cell cycle distribution was analyzed at the indicated time points using flow cytometry. The blots show the degree of protein depletion in this experiment. (**C**) Bar diagram of G1/G2 ratios derived from the analysis shown in (B).

Two important hallmarks of cells devoid of ATM are hypersensitivity to DSB-inducing agents and defective activation of the cell-cycle checkpoints by DSBs (61,62). Many DDR players show a similar effect on cell survival but less commonly interfere with cell-cycle checkpoint activation (49,57,63,64). PABPN1 depletion increased the sensitivity of cells to NCS or IR (Figure 3A, Supplementary Figure S3) and led to a striking cell cycle arrest at the G2/M boundary that persisted 48 hours after damage induction (Figure 3B and C). Collectively, the results substantiated our assumption that PABPN1 plays a role in the ATM-dependent response to DNA damage.

### APA is not globally modulated following DSB induction

RBPs may act in the DDR by binding and regulating the expression of pre-mRNAs that encode DDR players. It was previously reported that PABPN1 depletion resulted in global enhancement of cleavage and polyadenylation of proximal cleavage sites (CSs), which caused broad 3′UTR shortening at dozens of transcripts (43). We therefore sought to examine whether PABPN1 phosphorylation by ATM in response to DNA damage could also exert a global effect on APA. To examine this on a genomic scale, we first tested whether APA is modulated in response to DSB induction. We applied 3′-Seq, a deep-sequencing technique that allows precise mapping of 3′-end cleavage and polyadenylation sites at a nucleotide resolution (65) (Supplementary Figure S4). 3′-Seq also quantifies the usage of alternative CSs within 3′UTRs based on the number of reads that map to each of these sites, enabling detection of APA events induced under different biological conditions. Previous 3′-Seq studies demonstrated that, under highly proliferative conditions, usage of proximal 3′UTR CSs is significantly enhanced, resulting in global 3′UTR shortening (66–68), similar to the global effect observed in PABPN1-deficient cells (43,44).

**Figure 4.**
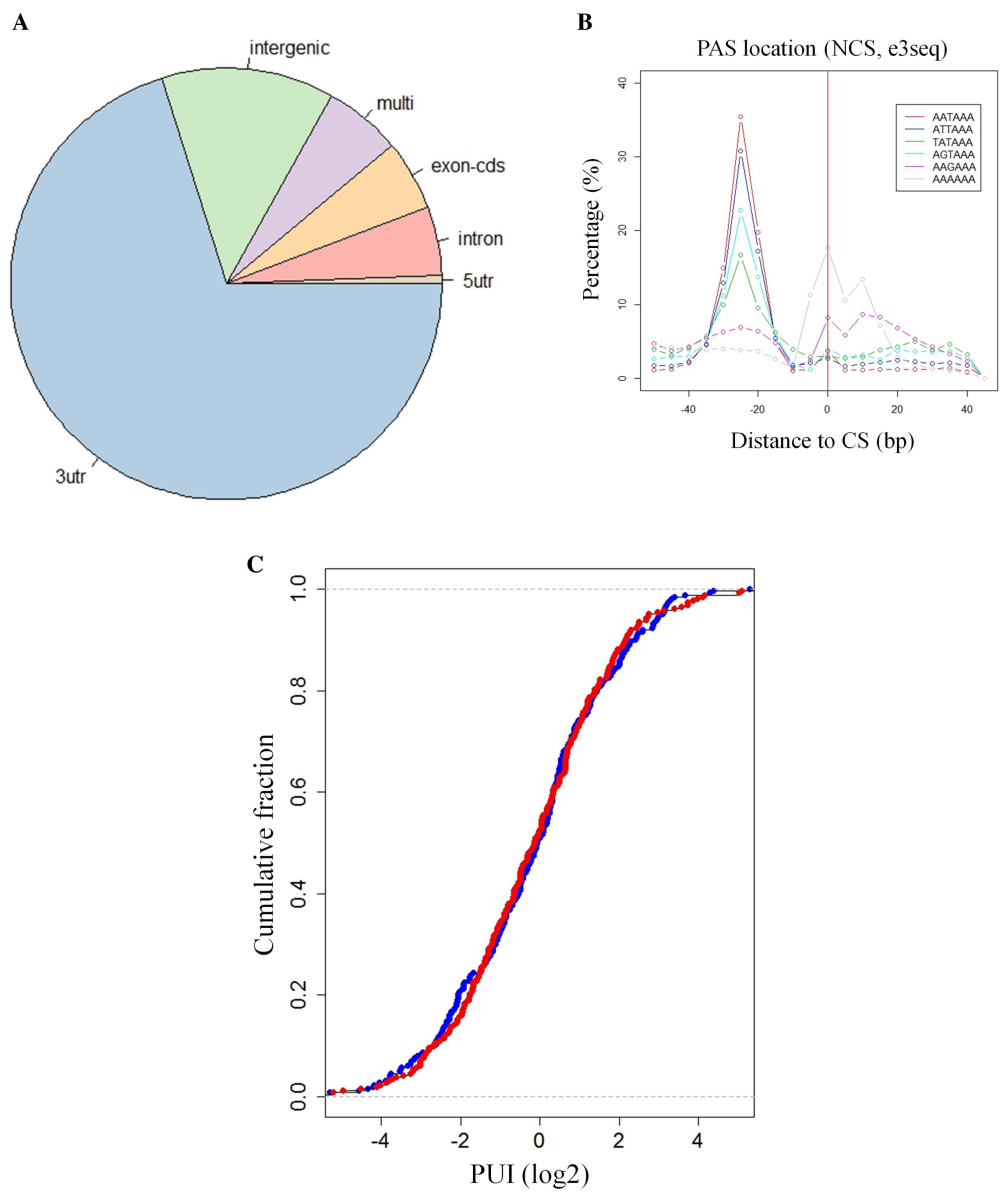
Global APA modulation is not detected following DSB induction. (**A**) Distribution of the CSs over different genome categories showed a highly significant enrichment for annotated protein-coding 3′UTRs. (**B**) As an additional indication of the precision of CS mapping by 3′-Seq, we searched for PAS signals (the canonical and its main variants) in ±100 nt with respect to the mapped CS. The PAS signals were significantly enriched at the correct location ∼20 nt upstream the CSs. (**C**) For each 3′UTR that showed a shift in CS usage upon NCS treatment, the usage of the proximal CS (relative to the usage of the other CSs in the same 3′UTR) was calculated. We call this relative usage Proximal Usage Index (PUI). A comparison of the distribution of PUIs between the NCS treated and control samples yielded no difference, indicating that there was neither global enhancement nor global reduction in usage of proximal CSs upon NCS treatment.

We applied 3′-Seq to U2-OS cells that had been treated with 20 ng/ml NCS and harvested 1, 2 and 4 h after treatment, and to untreated control cells harvested at the 4 h time point. We detected in this dataset 10131 putative poly(A) CSs; as expected, they were highly enriched for 3′UTRs (6411 sites mapped to annotated 3′UTRs of protein-coding genes) (Figure 4A). A further indication of the precision of the mapping of poly(A) CSs was the observation that the distribution of the canonical signal for cleavage and polyadenylation (AAUAAA) and its main variants, exhibited a very sharp peak at the expected location ∼20 nt upstream the cleavage site (CS) (Figure 4B).

Seeking modulation of APA upon DNA damage, we next identified 769 3′UTRs that contained more than one CS, and statistically tested each of them for alteration in the relative usage of its CSs in response to NCS. This analysis compared treated samples from each time point to the untreated control sample. The comparison between the NCS-4 h and control samples identified statistically significant shifts in CS usage in 263 3′UTRs (FDR<5%). When we examined whether those shifts showed any preference for proximal or distal CSs, there was no global 3′UTR shortening or lengthening in response to DSB induction: 55% and 45% of the 263 3′UTRs showed enhanced or reduced usage of the proximal CS, respectively, which is not significantly different from the 50%-50% ratio expected by chance (Figure 4C). Nor was any global effect observed at the other time points after NCS treatment. Since the dose of 20 ng/ml NCS is lethal, we examined whether APA might be globally modulated in response to lower doses. We were also interested in whether APA plays a role in late stages of the DDR. We therefore repeated the experiment using either 5 or 10 ng/ml of NCS and harvested the cells at 2, 6 and 24 h after treatment. Untreated control was harvested at 2 h. Here, too, no global effect was observed (data not shown). Collectively, these results indicate that there is no global enhancement or attenuation of usage of proximal CSs in response to NCS-induced damage and therefore, PABPN1’s function in the DDR is not associated with its role in regulation of global APA.

To further examine whether PABPN1 globally regulated pre-mRNA transcripts by a mechanism other than APA, in the context of DNA damage, we applied RNA-Seq to U2-OS cells depleted of PABPN1. 186 and 421 genes were up- or down-regulated upon PABPN1 depletion. We specifically sought an effect on the expression of DSB repair genes, and therefore searched the Gene Ontology (GO) lists for genes annotated as related to NHEJ and HRR (60 and 98 genes, respectively). Among those genes, only *BRCA1* showed ∼3-fold downregulation at the mRNA level in PABPN1-depleted cells (Supplementary Table S2). Correlatively, previous RNA-seq’ analysis performed in Hela cells depleted of PABPN1, demonstrated that 78 and 227 genes were up- or down-regulated. Here too, the only affected DSB repair-related gene was *BRCA1*, showing ∼2-fold down-regulation in terms of mRNA levels, upon PABPN1 depletion (69). To examine whether this regulation at the mRNA level had any impact on BRCA1 protein level, we examined its level in PABPN1-depleted cells using immunoblotting. Importantly, BRCA1 protein level was not affected in PABPN1-depleted cells (Supplementary Figure S5) Altogether, these results strongly suggest that PABPN1’s role in the DSB response is not related to regulation of the expression of DSB repair genes.

### Functional significance of PABPN1 phosphorylation in the DDR and its recruitment to sites of DNA damage

**Figure 5.**
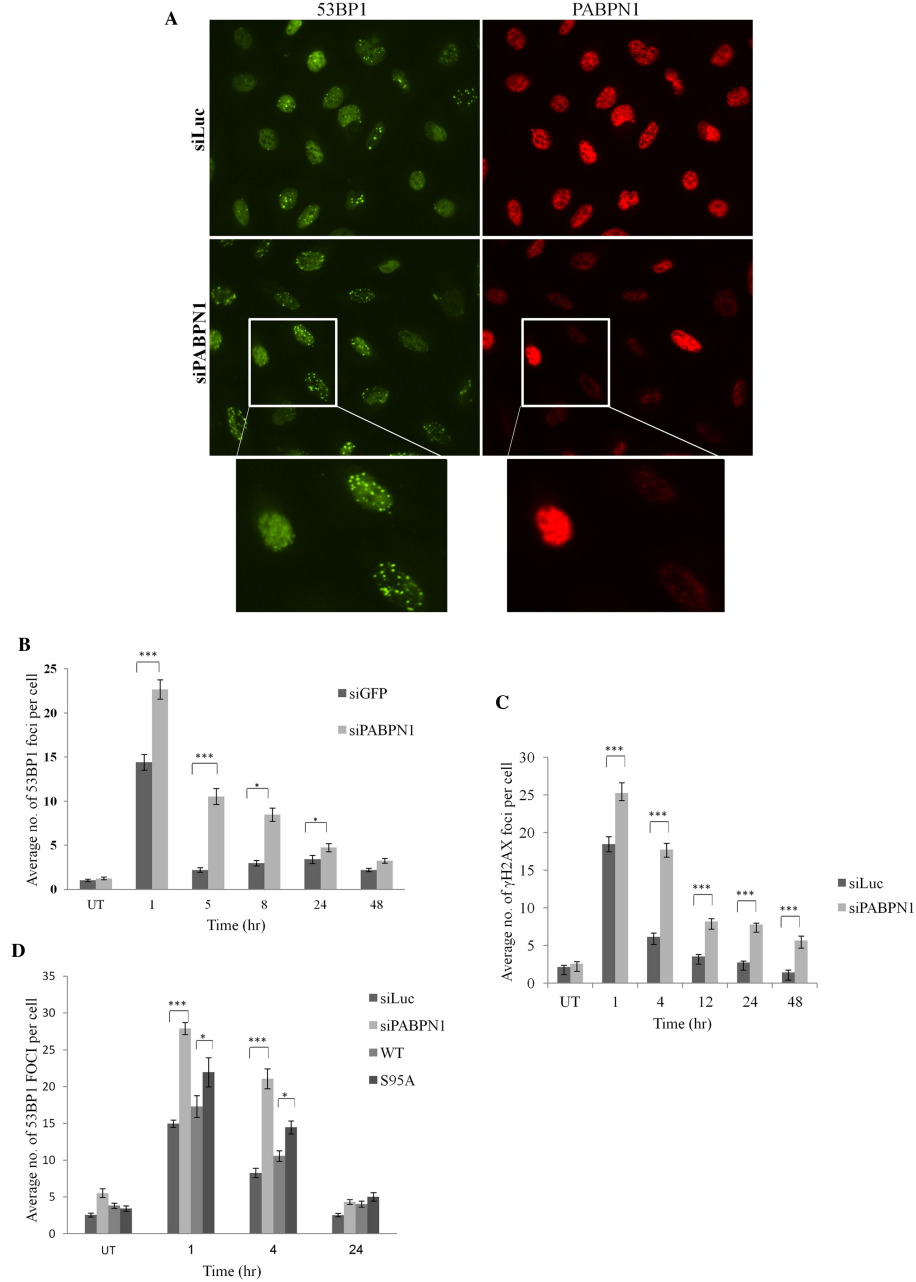
PABPN1 presence and phosphorylation are required for timely dissolution of NCS-induced 53BP1 nuclear foci. (**A**) U2-OS cells transfected with siLuc or siPABPN1 were treated with 3.5 ng/ml NCS, and co-immunostaining of PABPN1 and 53BP1 was carried out 8 h later. Note the striking difference between PABPN1-positive and -negative cells with regard to presence of 53BP1 foci. (**C**) Average counts of 53BP1 foci at various time points after treatment with 3.5 ng/ml NCS in PABPN1-proficient and -deficient cells (average of 100 cells). Bars represent SEM. Only cells negatively stained for PABPN1 were considered PABPN1-deficient. (**C**) Average counts of γH2AX nuclear foci at various time points after irradiation with 1 Gy IR in PABPN1-proficient and –deficient cells (average of 100 cells). Bars represent SEM. Only cells negatively stained for PABPN1 were considered PABPN1-deficient. (**D**) Similar analysis as in (**B**) of cells in which endogenous PABPN1 was depleted and cells in which it was replaced by ectopic, WT or S95A mutant PABPN1. The data represent five sets of experiments for 53BP1 and two sets for γH2AX. Error bars represent SEM. Statistical analysis was based on Student's *t* test.

**Figure 6.**
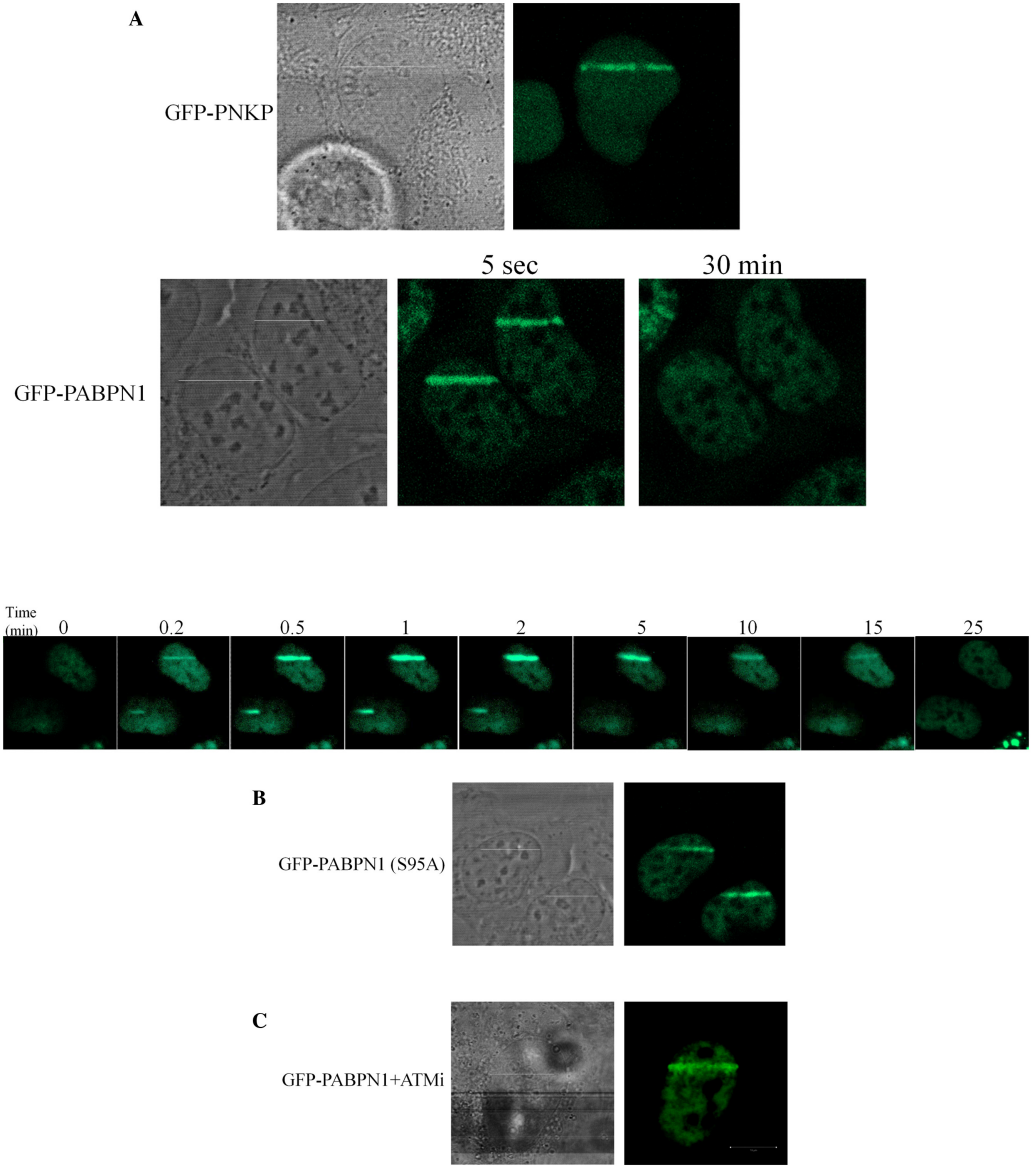
PABPN1 is physically recruited to sites of DNA damage. Live imaging snapshots demonstrate the accumulation of ectopic GFP-PABPN1 in WT (**A**) or S95A (**B**) form in areas of laser-induced DNA damage in U2-OS cells. Note the kinetics of PABPN1 recruitment to DNA damage sites (lower panel of A). (**C**) Lack of effect on PABPN1 recruitment of the ATM inhibitor, KU55993 applied at 10 μM 30 min prior to irradiation. Recruitment of GFP-tagged polynucleotide kinase-phosphatase (GFP-PNKP) (63,119) served as control for induction of DNA damage.

The above results suggested that, like many other DDR players, PABPN1 is borrowed temporarily from the RNA arena to serve the DDR. The next question was, whether PABPN1 functions in processes that take place at DSB sites. This could be reflected in abrogation of DSB repair upon PABPN1 depletion. We first monitored the dynamics of two hallmarks of unrepaired DSBs in PABPN1-depleted cells following DSB induction: nuclear foci of phosphorylated histone H2AX (γH2AX) (70) and the central DDR player, 53BP1 (71). PABPN1 depletion led to delayed disappearance dynamics of both DSB markers (Figure 5A–C). Importantly, expression of ectopic, WT PABPN1 rescued this phenotype, but the S95A mutant protein failed to fully rescue it (Figure 5D), suggesting that the phosphorylation at Ser95 was functionally important in this regard. Further indication that PABPN1 functions in the DDR context at the sites of DNA damage could be its relocalization to these sites. We induced spatially localized DNA damage using a focused laser beam and monitored the localization of ectopic GFP-tagged PABPN1. A distinct fraction of GFP-PABPN1 was recruited to the stripes of laser-induced damage within a few seconds of DNA damage induction, culminated within a few minutes (Figure 6A) and remained there for ∼30 min. Importantly, non-phosphorylatable PABPN1 was similarly recruited to laser stripes, and inactivation of ATM using the ATM inhibitor did not interfere with this process (Figure 6B and C), indicating that ATM-dependent phosphorylation of PABPN1 as well as of other ATM targets was not required for PABPN1 recruitment. Collectively, the results suggested that PABPN1 is physically and functionally recruited by the DDR and plays an important role in the early phases of the DSB response, and subsequently-in DSB repair.

### PABPN1 is required for the function of DSB repair pathways

**Figure 7.**
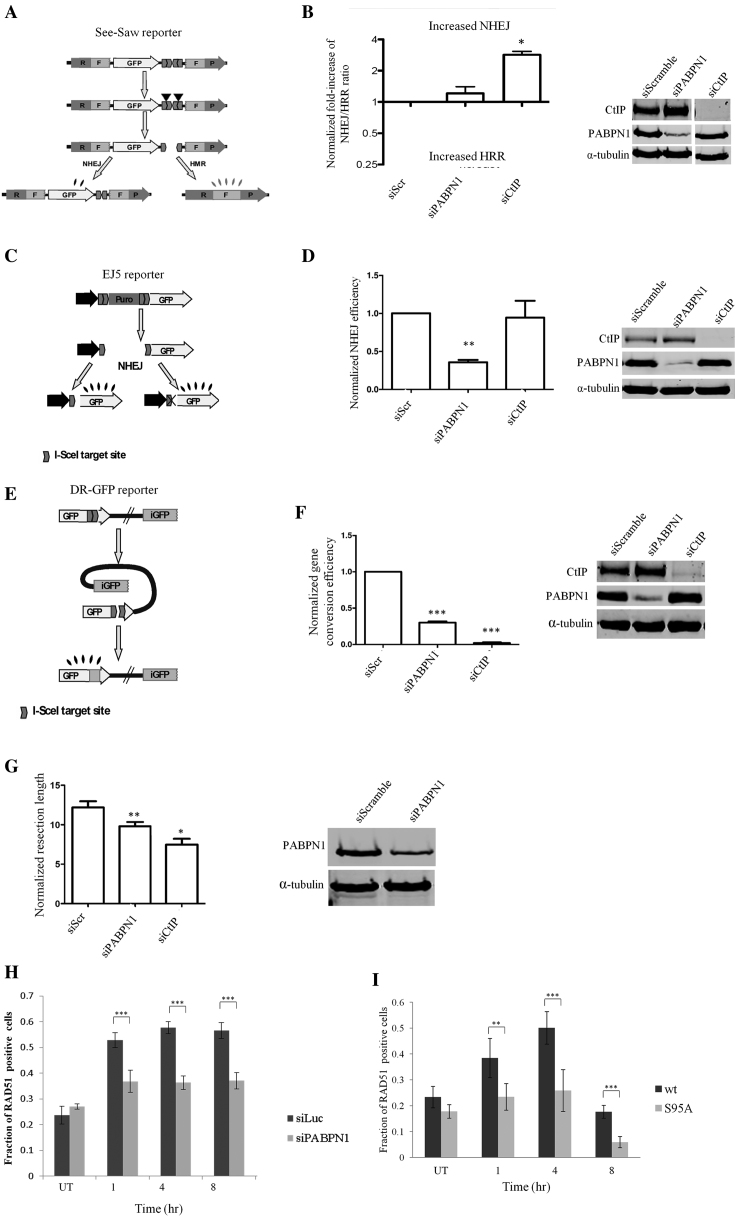
PABPN1 is required for optimal DSB repair. (**A, B**) Effect of PABPN1 depletion on NHEJ/HRR ratio. Cells were transfected with the indicated siRNAs and the SSR 2.0 assay was applied (52). The ratio of green to red cells was calculated and normalized for each siRNA against the effect of a scrambled siRNA (siScr). An NHEJ/HRR ratio different from the baseline value of 1.0 indicates an imbalance between the two DSB repair pathways. Depletion of the protein CtIP, which is essential for proper end-resection (120) served as a positive control. Bars represent the average and standard deviation of three independent experiments. (**C, D**) PABPN1 is necessary for efficient C-NHEJ. The EJ5 reporter (72) is constructed such that I-SceI-induced DSB can be repaired by NHEJ, recreating an active GFP gene that does or does not contain a functional I-SceI target site. The percentage of green cells, calculated as described in (51), was normalized against cells transfected with a control siRNA. (**E, F**) PABPN1 is required for proper HRR. The DR-GFP reporter (50) is formed by two non-functional copies of the GFP. Gene conversion induced by an I-SceI-mediated DSB restores an active GFP gene. The efficiency of gene conversion was calculated as described for NHEJ. (**G**) PABPN1 is required for efficient DNA-end resection at DSB sites. Single Molecule Analysis of Resection Tracks (SMART) analysis (54) of cells depleted of PABPN1 or the end-resection regulator CtIP. The length of individual fibers was measured and the median of at least 250 fibers was calculated. (siScr = siScramble). (**H**) PABPN1 depletion impairs the assembly of RAD51 foci at DSB in response to DSB induction. U2-OS cells transfected with siRNAs against Luciferase (control) or PABPN1 were treated with 3.5 ng/ml NCS, and stained at the indicated time points for RAD51. Bars represent percentage of RAD51 positive cells (a minimum of 200 cells were counted). The data represent three sets of experiments. Error bars represent SEM. Statistical analysis was based on chi-square test. (**I**) Similar analysis as in (H) of cells in which endogenous PABPN1 was depleted and cells in which it was replaced by ectopic, WT or S95A mutant PABPN1.

The delicate balance between DSB repair pathways is tightly regulated and is crucial for genome integrity (12). We used a recently established assay to measure the ratio between the two main DSB repair pathways, C-NHEJ and HRR (52) in cells depleted of PABPN1. Briefly, the SeeSaw reporter (Figure 7A), which harbors a CS of the I-SceI nuclease, is integrated in the genome of U2-OS cells. Cleavage by I-SceI induces repair by C-NHEJ, which is measured by GFP fluorescence, or by HRR, measured by RFP fluorescence. The GFP/RFP ratio is indicative of the balance between the two repair pathways. PABPN1 depletion had no significant effect on this ratio (Figure 7B). However, when the involvement of PABPN1 in each of the pathways was examined separately using well-documented reporters (Figure 7C and E) (50,51,72), cells devoid of PABPN1 clearly manifested faulty repair in both pathways (Figure 7D and F). Since HRR operates in the late S and G2 phases of the cell cycle, disruption of normal cell cycle dynamics can interfere with the NHEJ:HRR balance. Notably, the deficiency in HRR was evident despite the marked G2/M arrest observed in PABPN1-depleted cells following NCS treatment (Figure 3B), reinforcing the role of PABPN1 in the HRR pathway. The sub-optimal repair through HRR can be ascribed to a defect in deep 5′ to 3′ DNA end-resection at DSB ends – a prerequisite for HRR (73). To determine whether PABPN1 plays a role in this process we utilized the recently developed SMART (Single Molecule Analysis of Resection Tracks) assay that measures the length of resected DNA at the level of single molecules (54). Importantly, PABPN1 knockdown significantly diminished the length of resected ends in IR-treated cells (Figure 7G). To appraise PABPN1’s involvement in HRR, we quantified the nuclear foci of a major HRR player, RAD51, which coats DNA single strand stretches following the deep end-resection-a vital step for the initiation of strand invasion in the homologous recombination process (10). Notably, the fraction of cells with RAD51 foci in PABPN1-deficient cells was significantly lower than in PABPN1-proficient cells (Figure 7H). Importantly, expression of ectopic, WT PABPN1 rescued this phenotype, but the S95A mutant protein failed to rescue it (Figure 7I), suggesting that the phosphorylation at Ser95 was functionally significant in this regard. Altogether, these results firmly establish a role for PABPN1in the regulation of the two major DSB repair pathways.

### Dynamics of PABPN1’s protein-protein interactions following DNA damage induction substantiates its involvement in the DDR

DNA damage-induced protein–protein interactions are central to the DDR. DSB-induced PABPN1 interacting proteins were searched for by immunoprecipitation of total-PABPN1 or phospho-PABPN1 followed by mass spectrometry. Results are presented in Supplementary Table S3. We assume that the functional importance of PABPN1 in the DDR is associated with its phosphorylated portion, which we found to be small (Figure 1C). We therefore expected to capture valuable DNA damage-induced interactions of PABPN1 by using the anti-pPABPN1 antibody for immunoprecipitation.

**Figure 8.**
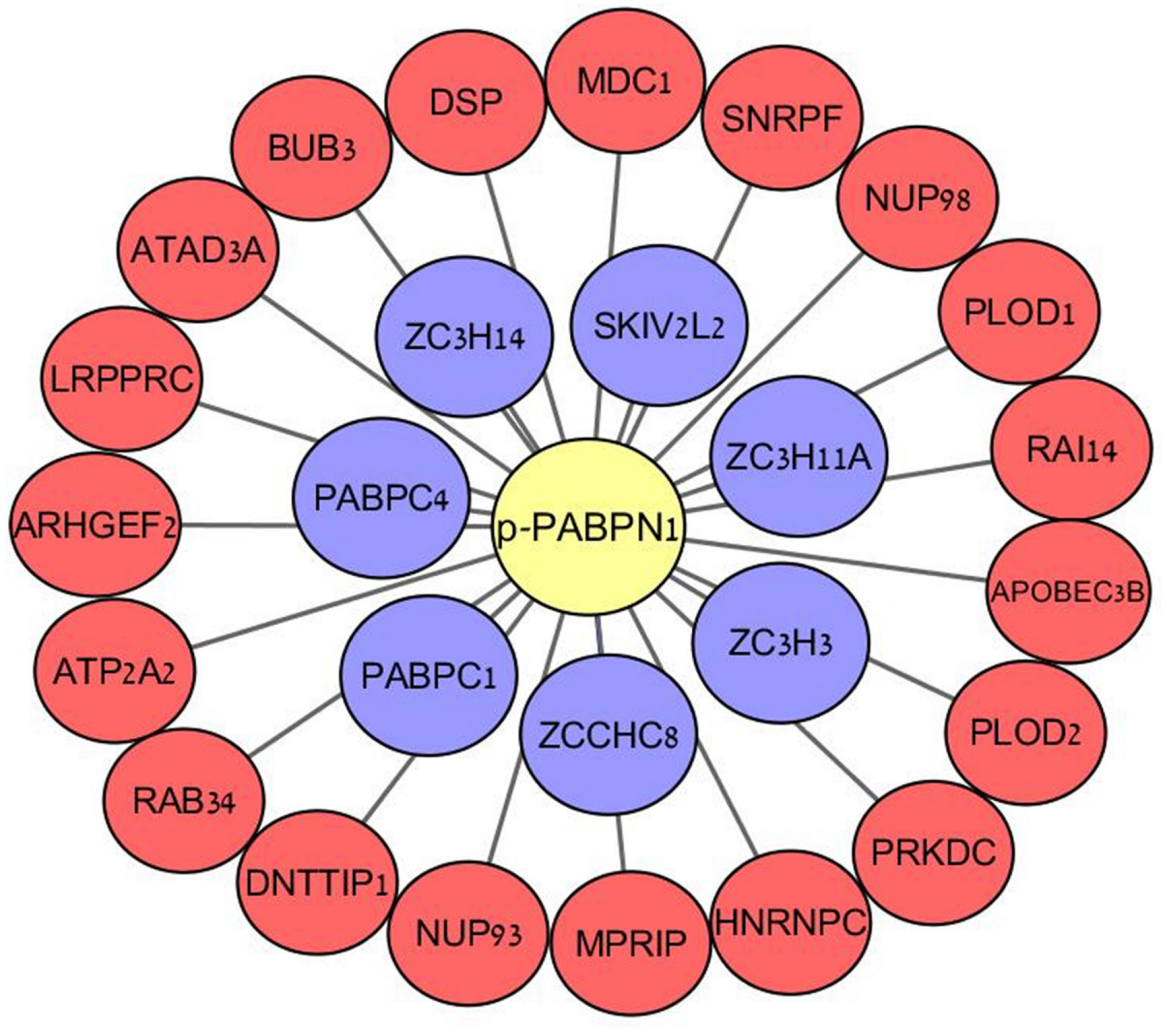
Protein-protein interactions of phospho-PABPN1 following induction of DNA damage. U2-OS cells were treated with 50 ng/ml NCS for 30 min and cell lysates were used for immunoprecipitation using antibodies against pPABPN1 or total PABPN1. The immune complexes were subjected to mass spectrometry analysis. The specific interactors of pPABPN1 are presented. Red nodes – proteins that precipitated only with phospho-PABPN1. Blue nodes—proteins that precipitated with both phospho- and total-PABPN1.

We identified a total of 26 proteins that co-immunoprecipitated with pPABPN1 after NCS treatment (Figure 8, Table 1). Of these, 7 are 3′-end pre-mRNA processing and polyadenylation factors, most of which were previously shown to interact with PABPN1 as part of the 3′-end pre-mRNA processing complex in unperturbed cells. Indeed, these proteins were also immunoprecipitated with total-PABPN1 in untreated and NCS-treated cells (Figure 8, blue nodes). It will be interesting to see if any of these proteins belongs to a functional module that includes PABPN1 and is recruited by the DDR. Importantly, the other 19 proteins that interact with pPABPN1 following induction of DNA damage include two well-established DDR players -DNA-PKcs (PRKDC) and MDC1, corroborating the functional significance of PABPN1 in the DDR. An intriguing interactor of pPABPN1 is the BUB3 mitotic checkpoint protein. BUB3 is a member of the mitotic checkpoint complex (MCC, composed also of MAD2 and BUBR1), which was recently shown to be regulated by ATM and MDC1 (74). This interesting interaction may provide insights into the role of PABPN1 in the damage-induced G2/M cell-cycle checkpoint. Other proteins in this group are associated with RNA metabolism: most notably the promiscuous RBP hNRNPC, which is highly prevalent as a recurrent hit DDR screens (for instance, (28–31,75–77)), which functions there in its capacity as regulator of mRNA expression (78,79). Likewise, SNRPF, a core component of the spliceosome that regulates pre-mRNA splicing, was recently identified in a DDR screen to be targeted by ubiquitination following both IR and UV treatment (80,81). Other notable interactors include NUP98 and NUP93—members of the nuclear pore complexes (NPCs). NPCs play a role in posttranscriptional regulation by selectively controlling the translocation of nuclear mRNA into the cytoplasm, where translation subsequently takes place (82). Interestingly, NUP98 and NUP93 were previously identified in proteomic screens to be phosphorylated, dephosphorylated and ubiquitylated following DNA damage induction (80,83,84). Another interactor linked to the RNA field is the RBP LRPPRC, which also turned up in various screens for new DDR players (80,85,86). The precise role of LRPPRC is unknown, but studies indicate that it functions in different aspects of RNA metabolism, especially in transcriptional regulation of both nuclear and mitochondrial genes (87–91). Collectively, these results may point to a suit of RNA metabolism players which, together with PABPN1, are physically and functionally recruited by the DDR from their regular context.

**Table 1.** List (A–Z) of PABPN1 protein–protein interactors (see Figure 8). Hits in previous screens for DDR players are indicated. In red - proteins that co-precipitated with pPABPN1 after NCS treatment. In blue: proteins that co-precipitated with pPABPN1 after NCS treatment and also with total PABPN1 in untreated and in NCS-treated cells

Gene name	Description	Gene id	Hits in previous screens for DDR players (refs.)
APOBEC3B	Apolipoprotein B MRNA Editing Enzyme Catalytic Subunit 3B	9582	(28,80)
ARHGEF2	Rho/Rac Guanine Nucleotide Exchange Factor 2	9181	(80,121)
ATAD3A	ATPase Family, AAA Domain Containing 3A	55210	
ATP2A2	ATPase Sarcoplasmic/Endoplasmic Reticulum Ca2+ Transporting 2	488	(80,81,122)
BUB3	BUB3, Mitotic Checkpoint Protein	9184	(29,75,80,81)
DNTTIP1	Deoxynucleotidyltransferase Terminal Interacting Protein 1	116092	(80,81)
DSP	Desmoplakin	1832	(30,80,81,84)
HNRNPC	Heterogeneous Nuclear Ribonucleoprotein C (C1/C2)	3183	(27–31,75–77,80,81,123,124)
LRPPRC	Leucine Rich Pentatricopeptide Repeat Containing	10128	(80,86,121)
MDC1	Mediator Of DNA Damage Checkpoint 1	9656	(30,31,77,80,81,83,84,121,125,126)
MPRIP	Myosin Phosphatase Rho Interacting Protein	23164	(84)
NUP93	Nucleoporin 93	9688	(80,81,83,84)
NUP98	Nucleoporin 98	4928	(80,84)
PLOD1	Procollagen-Lysine,2-Oxoglutarate 5-Dioxygenase 1	5351	
PLOD2	Procollagen-Lysine,2-Oxoglutarate 5-Dioxygenase 2	5352	(29,30)
PRKDC	DNA-PK Catalytic Subunit	5591	(30,31,76,77,80,81,83,84,127)
RAB34	RAB34, Member RAS Oncogene Family	83871	(80,81,84,128)
RAI14	Retinoic Acid Induced 14	26064	(27,80,83,84)
SNRPF	Small Nuclear Ribonucleoprotein Polypeptide F	6636	(80,81)
PABPC1	Poly(A) Binding Protein Cytoplasmic 1	26986	(28,30,75,80,129,130)
PABPC4	Poly(A) Binding Protein Cytoplasmic 4	8761	(80,128,129)
SKIV2L2	Ski2 Like RNA Helicase 2	23517	(30,80)
ZC3H11A	Zinc Finger CCCH-Type Containing 11A	9877	(30,31,80,84,131)
ZC3H14	Zinc Finger CCCH-Type Containing 14	79882	(27,30,31,80,83)
ZC3H3	Zinc Finger CCCH-Type Containing 3	23144	(84)
ZCCHC8	Zinc Finger CCHC-Type Containing 8	55596	(30,81)

## DISCUSSION

Numerous cellular physiological pathways are temporarily integrated into the DDR, contributing to the rapid formation of this elaborate network upon induction of DNA damage. The physical proximity of RBPs to chromatin together with their multiple capacities makes them accessible and interesting candidates for such temporary integration in the DDR network. The emerging role of RBPs in the DDR is reflected in the steep rise of the appearance of these proteins as hits in proteomic and functional screens for DDR players (27–31). Indeed, the awareness to this growing interface between the RNA metabolism and genome stability arenas is rapidly growing (32,33,36) and it has become evident that RBPs can potentially act in the vicinity of DNA damage sites or remotely by altering gene expression (32–38). A recent interesting example obtained in yeast is the involvement of *S. cerevisiae* RNA decay factors in regulation the process of single-strand DNA coating by replication protein A, at DSB sites—a critical process in DSB repair (92). Notably, several established DDR players have an RNA binding capacity, including 53BP1, KU70, KU80, ATR and BRCA1 (93–97), though the functional significance of this capability in the DDR is still unclear. An important facet of the RNA-DDR link is the recent discovery of the production of small, non-coding RNA molecules at DSB sites, which are required for optimal activation of the DDR (98–101).

Screens searching for ATM targets identified RBPs as important modules in the ATM-mediated DDR network (30,31), but few studies have focused on detailed analysis of ATM-dependent PTMs of individual RBPs. We were therefore intrigued by the identification of PABPN1 as a hit in our phosphoproteomic screen (31). Our results firmly establish PABPN1 as a novel target of ATM in response to DSBs and a new player the delicate regulation of DSB repair pathways. The dynamics of its ATM-mediated phosphorylation is typical of many ATM targets, and our results attest to the functional importance of this phosphorylation.

PABPN1-depleted cells exhibited both radiomimetic hypersensitivity, which usually indicated faulty DSB repair, and a pronounced G2/M cell-cycle arrest in response to DSB induction - two characteristics of cells devoid of ATM (61,62). A possible link between PABPN1 and cell cycle control may come from its possible interaction with the BUB3 mitotic checkpoint protein, suggested by our proteomic results. BUB3 is a member of the mitotic checkpoint complex (MCC), which is the central activator of the mitotic spindle assembly checkpoint (SAC). The SAC controls metaphase-to-anaphase transition by guaranteeing the fidelity of chromosome segregation (102–104). Intriguingly, ATM and MDC1 were recently shown to control the formation of an intact MCC during SAC activation (74). It was demonstrated that H2AX is phosphorylated at mitotic kinetochores by ATM upon SAC induction, and MDC1 interacts with the MCC in a manner dependent on this phosphorylation. Interestingly, the scaffold DDR protein, MDC1, appeared among the potential binding partners of pPABPN1 in our proteomic analysis. Possible functional interaction among pPABPN1, BUB3 and MDC1 may provide insights into the prolonged G2/M cell cycle arrest observed in PABPN1-deficient cells following DSB induction.

Our initial interest in PABPN1 as potential DDR player stemmed from its role in a global process affecting the expression of many genes—APA (43–45). Our hypothesis was that ATM might regulate APA by phosphorylating PABPN1. Our first question was, whether APA was indeed modulated in response to DNA damage. In two independent large-scale 3′-RNA-seq' experiments we found that APA was not globally modulated in NCS-treated cells. While our experiments point to some individual pre-mRNA transcripts that might be regulated by APA in response to DNA damage, the absence of a global and consistent trend in regulated transcripts (3′UTR shortening or lengthening) makes tracking them somewhat arbitrary. Furthermore, taking into account the global effect of PABPN1 on APA suppression (i.e. strong 3′UTR shortening in PABPN1-depleted cells), we would expect that if such regulation existed in the context of DNA damage, it too would manifest globally.

The role of PABPN1 in global regulation of pre-mRNA transcripts reaches beyond the scope of APA, as evidenced by its longstanding role in poly(A) tail binding of all nascent pre-mRNAs (40–42,105,106). The poly(A) tail influences many aspects of mRNA metabolism, including subcellular localization, mRNA stability and translation efficiency (107,108). Furthermore, PABPN1 globally regulates the expression of non-coding RNAs (ncRNAs), including long non-coding RNAs (lncRNAs) and small nucleolar RNAs (snoRNAs) (69,105,109,110), the importance of which is increasingly evident in the DDR (36,37,111). Nevertheless, RNA-seq analysis in PABPN1-depleted cells performed by us and others indicate that PABPN1s role in the DDR is probably not related to pre-mRNA expression regulation. Yet, we cannot rule out the possibility that PABPN1 globally regulates mRNA stability and/or ncRNAs by a mechanism other than APA in the context of DNA damage.

We were, however, able to show clearly PABPN1’s role in the regulation of DSB repair, and its physical recruitment to DSB sites, a typical characteristic of proteins that function in this capacity. Intriguingly, PABPN1 is important for optimal function of both NHEJ and HRR. Previously, we found that the monoubiquitylation of histone H2B at DSB sites was important for timely function of both repair pathways (49). The concomitant effect on both processes could be explained by the role of H2B monoubiquitylation in a basic process that is critical for DSB repair—relaxation of the 30 nm chromatin fiber (112). PABPN1’s function in NHEJ might be associated with its physical association with DNA-PKcs, suggested by our proteomic analysis. Notably, this is not the first time that DNA-PK and PABPN1 are involved in the same process. PABPN1 is associated with RNA polymerase II (RNAP II) during transcription and is thought to take part in the kinetic coupling between 3′-end pre-mRNA formation and transcription termination (113,114). Interestingly, it was recently demonstrated that NHEJ proteins, including DNA-PKcs, form a multiprotein complex with RNAP II (115) and the arrest of RNA polymerase II transcription, which occurs concomitant to DSB induction, is DNA-PK-dependent (38,116). Notably, DNA-PK and PABPN1 both arrive quickly at DSB sites. Moreover, it has been recently shown that C-NHEJ repairs transcribed genes and that nascent pre-mRNA are part of the NHEJ complex and might serve as a template to retrieve missing information through error-free end joining (115). Hence, it is possible that the role of PABPN1 in pre-mRNA metabolism is utilized to process such RNAs in the DDR. Interestingly, a recent study demonstrated that transcript RNA is also used as template for DSB repair in HRR pathway (117). As for HRR, the marked cell-cycle arrest at the G2/M boundary in cells depleted of PABPN1 substantiates the finding of defective HRR in the absence of this protein. We found that PABPN1 is required for efficient DNA end-resection at DSB ends, an initial key step in this pathway. Few other RNA binding factors such as hnRNPU-like proteins were shown to be required for proficient DNA-end resection (118). Indeed, another possible PABPN1 interactor that we identified is the prominent RBP, hNRNPC, which is recruited to DNA damage sites as part of the BRCA1/BRCA2/PALB2 complex and is required for correct pre-mRNA splicing and elevated expression of several HRR factors, including BRCA2, BRCA1, and RAD51 (78,79). PABPN1 and hnRNPC are both nuclear RBPs containing an RNA recognition motif (RRM), acting in similar facets of RNA metabolism. It would be interesting to investigate whether PABPN1 interacts with the BRCA1/BRCA2/PALB2 complex, and whether its involvement in the HRR pathway is associated with its physical link to hNRNPc. Furthermore, the identification of PABPN1 as an ATM target and a crucial player in the cellular response to DSBs establishes a novel ATM dependent-link between RNA binding proteins and the DDR.

## AVAILABILITY

Mass-spectrometric data are available via ProteomeXchange with identifier PXD005913.

## Supplementary Material

Supplementary Data
