# Supplementary material for: Nuclear poly(A)-binding protein 1 is an ATM target and essential for DNA double-strand break repair
Source: Nucleic Acids Res. 2017 Dec 14;46(2):730–47. doi: 10.1093/nar/gkx1240 (PMC5778506; doi:10.1093/nar/gkx1240)
Supplement: Supplementary Data [file gkx1240_supp.zip › nar-00116-d-2017-File016.docx]

**Supplementary data**

**
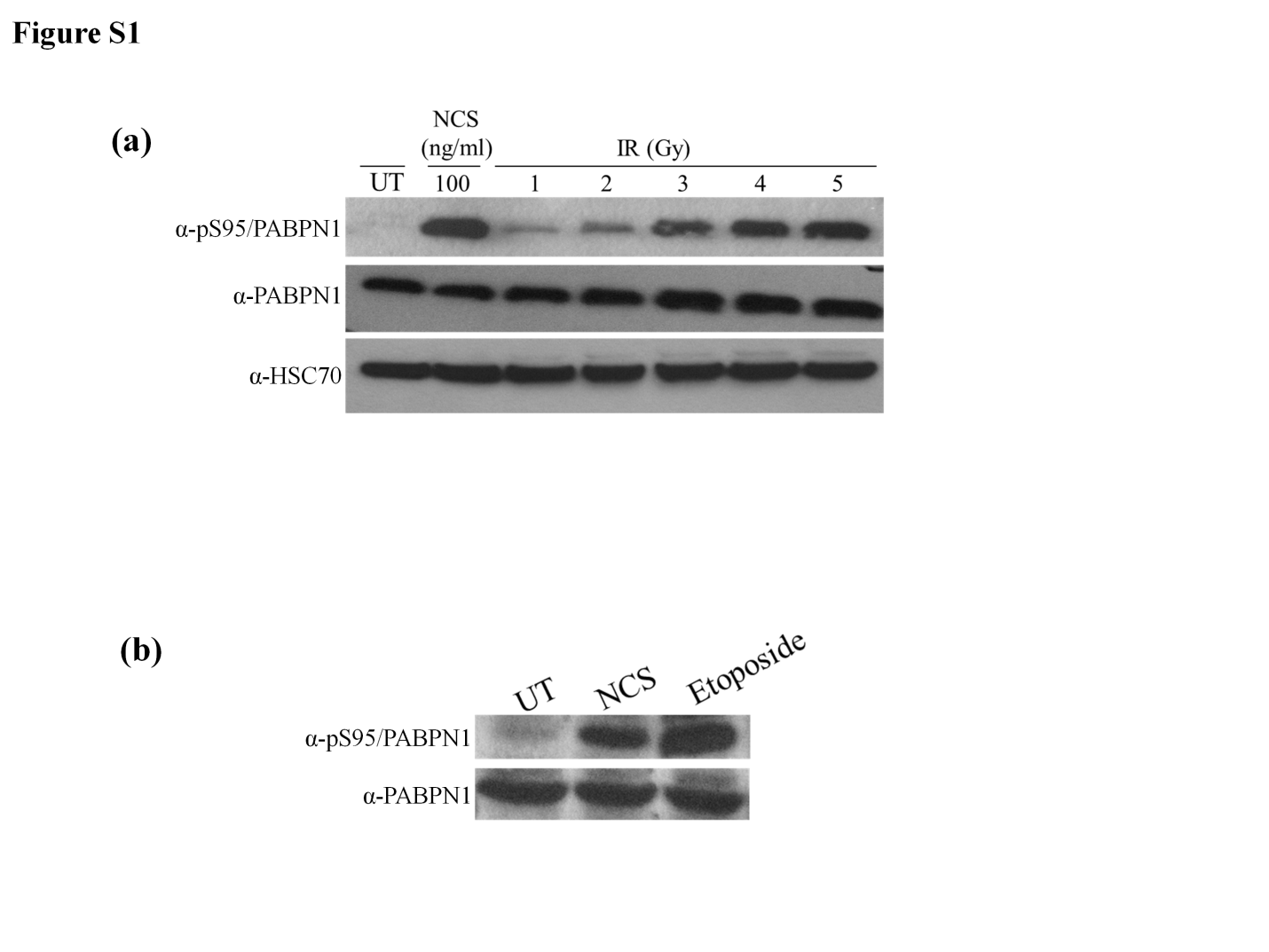
**

**Figure S1.** **PABPN1 phosphorylation is common to different forms of DSB induction**. Immunoblotting analysis of PABPN1 phosphorylation was carried out in cellular extracts of U2-OS cells treated with IR or etoposide. (a) Cells were treated with the indicated doses of IR and harvested 1 hr later. Cells treated with 100 ng/ml NCS served as a positive control for PABPN1 phosphorylation. (b) Cells were treated with 100 ng/ml NCS or 20 µM etoposide and harvested 2 hr later.

**
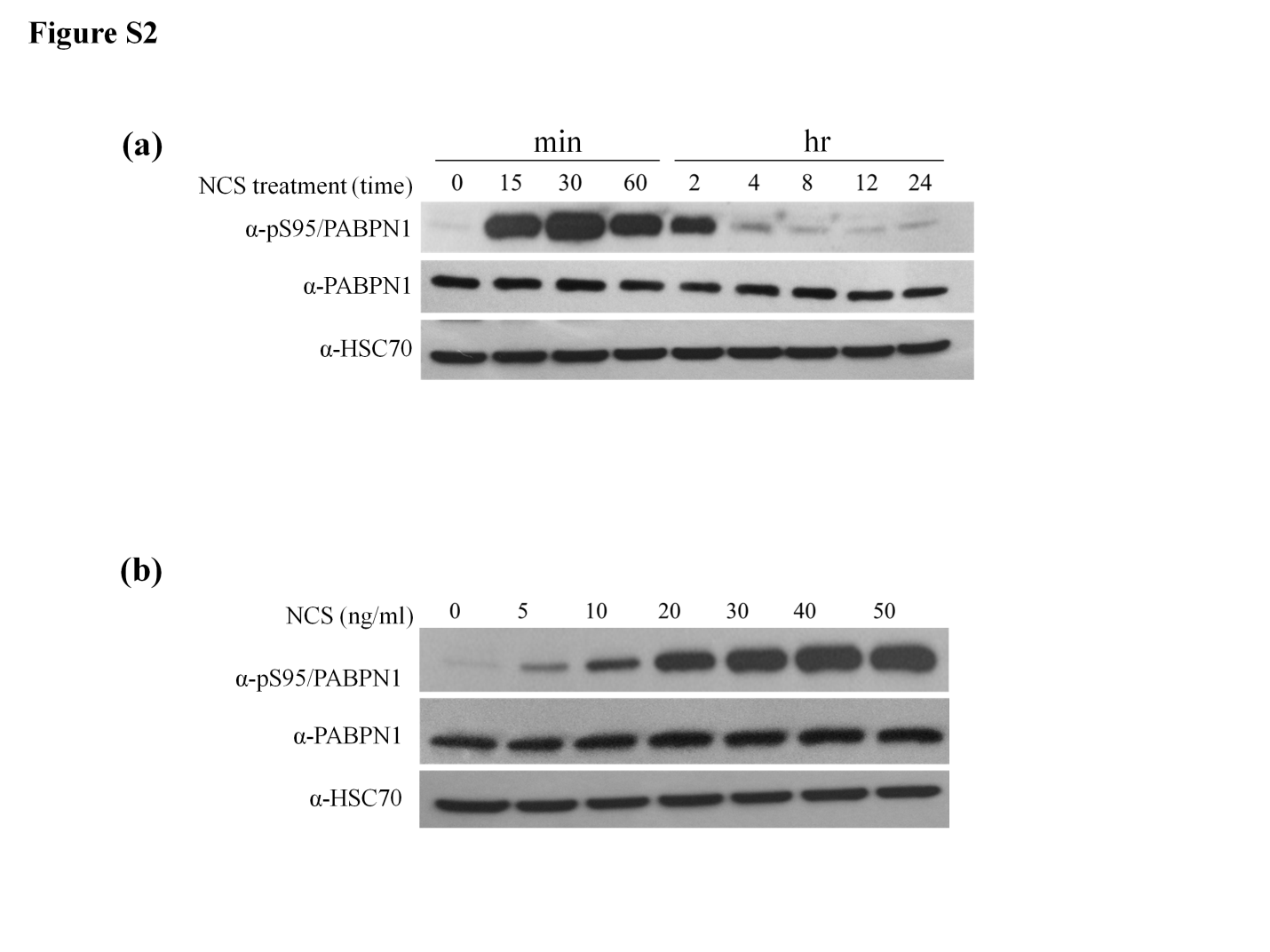
**

**Figure S2.** **Western blotting analysis presents dose response and time course of PABPN1 phosphorylation on Ser95 following DSB induction in U2-OS cells.** (a) Cells were treated with 20 ng/ml NCS and harvested at different time points after treatment. (b) Cells were treated with the indicated doses of NCS and harvested 1 hr later.

**
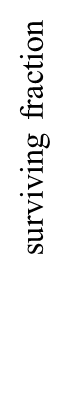
**

IR dose (Gy)

**
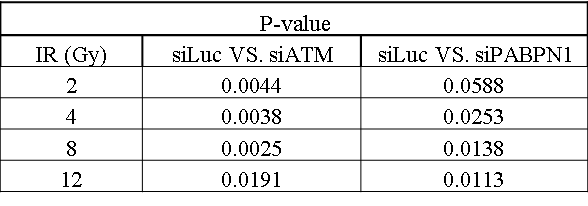
**

**
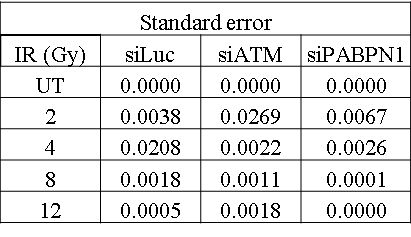
**

**Figure S3.** PABPN1-depleted cells exhibit IR hypersensitivity. Shown are survival curves based on clonogenic growth of HeLa cells transfected with siRNA against Luciferase (control), or ATM or PABPN1, and treated with increasing doses of the IR. The experiment was carried out in triplicates. Statistical analysis was based on Student’s t test. Tables below present p-values and SEM.

**
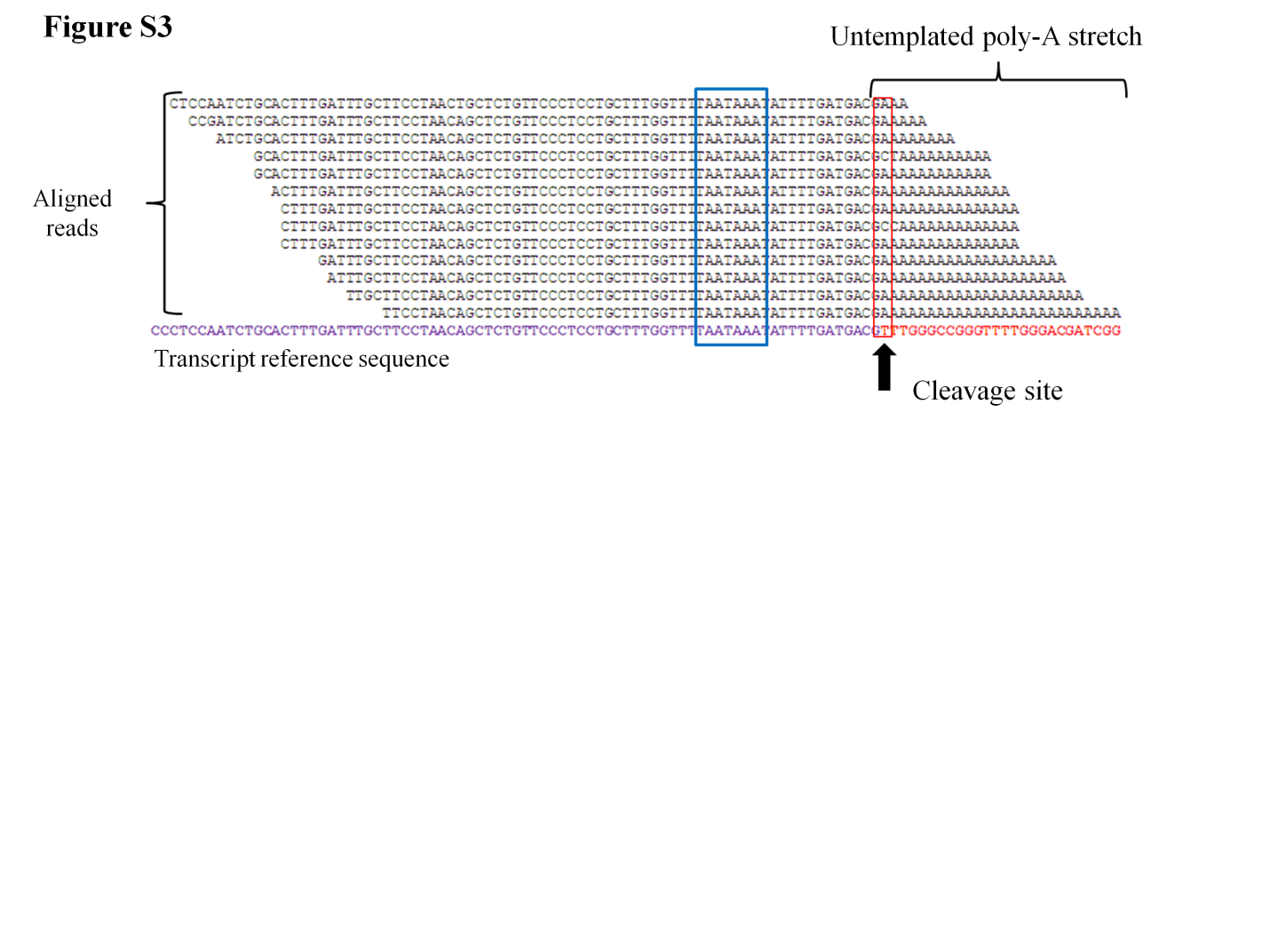
**

**Figure S4.** Example of reads sequenced by 3'seq and span the poly(A) CS, and therefore contain a stretch of As at their end that is not encoded in the genome. Such reads allow the precise mapping of the CS at nucleotide resolution. (Note the canonical PAS signal AATAAA upstream the mapped CS.)


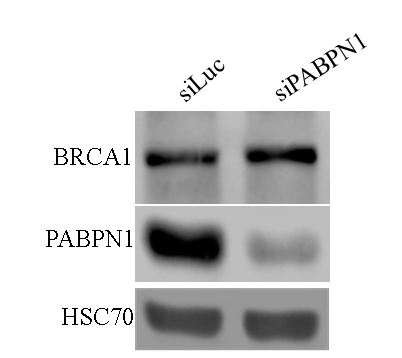


**Figure S5.** Detection of BRCA1 level in PABPN1 -depleted or -proficient U2-OS cells. Cellular extracts from PABPN1-depleted or -proficient U2-OS cells or ATM-depleted cells were subjected to immunoblotting analysis using the indicated antibodies. Three independent experiments were performed. A representative blot is shown

**
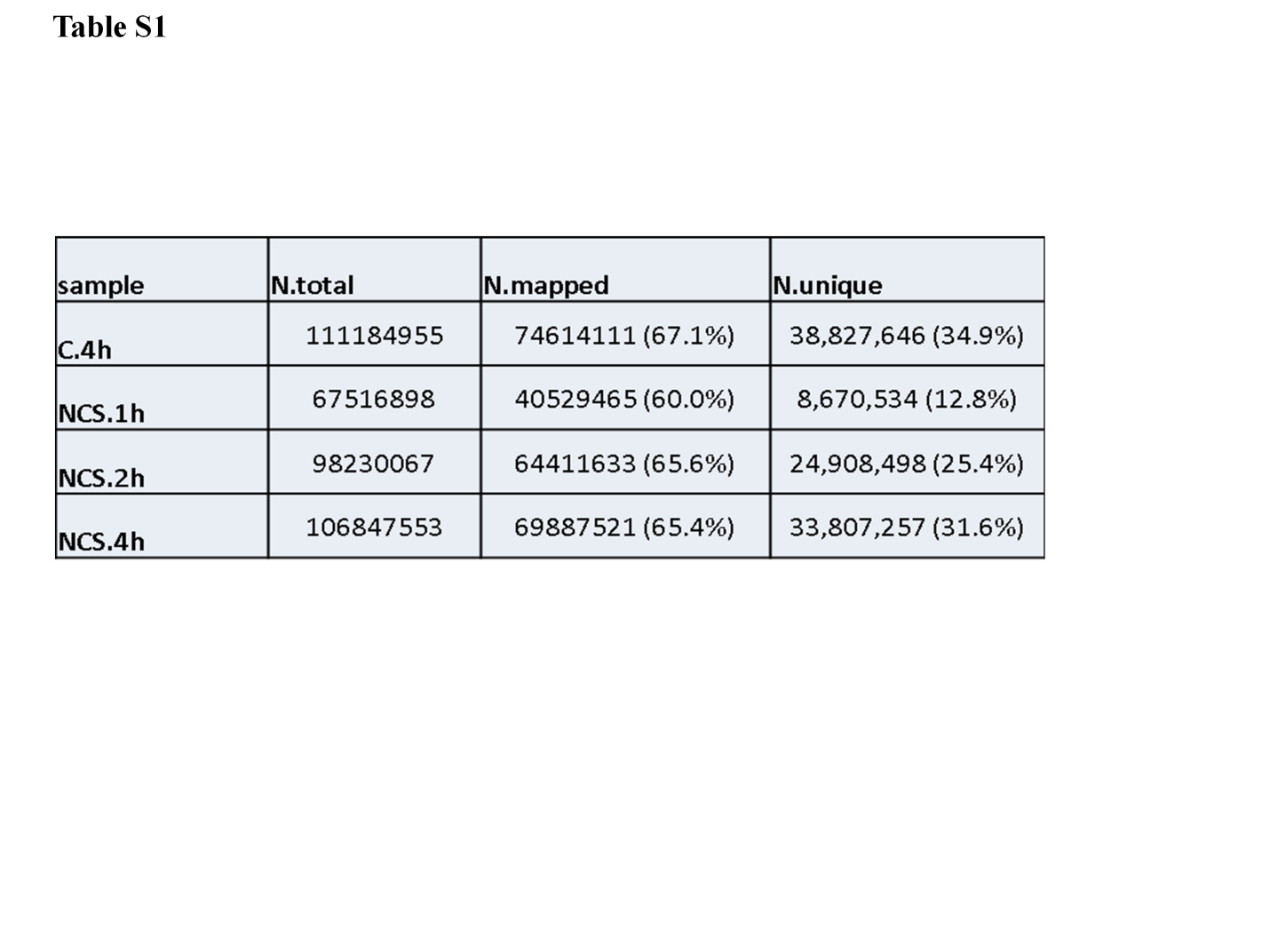
**

**Table S1.** Sequenced reads aligned to the human genome. Percentages of mapped reads (N.mapped) out of total reads (N.total) and only uniquely mapped reads out of total reads (N. unique) which were included in subsequent analysis.

**Table S2. Gene expression level in PABPN1-depleted and control U2OS cells measured by RNA-Seq.**

Added separately as an Excel file.

(a)

| **Gene name** | **Description** | **Gene id** | **Protein function** | **Hits in previous screens for DDR players (refs.)** |
| --- | --- | --- | --- | --- |
| APOBEC3B | Apolipoprotein B MRNA Editing Enzyme Catalytic Subunit 3B | 9582 | A member of the cytidine deaminase gene family. Acts as an inhibitor of retrovirus replication and retrotransposon mobility via deaminase-dependent and -independent mechanisms. Among its related pathways are RNA editing, cell growth and cell-cycle control. | ([1](#_ENREF_1),[2](#_ENREF_2)) |
| ARHGEF2 | Rho/Rac Guanine Nucleotide Exchange Factor 2 | 9181 | Activates Rho-GTPases by promoting the exchange of GDP for GTP. Involved in numerous cellular processes that are initiated by extracellular stimuli, including cell- cycle regulation, activation of NF-kappaB pathway and the innate immune response. | ([2](#_ENREF_2),[3](#_ENREF_3)) |
| ATP2A2 | ATPase Sarcoplasmic/Endoplasmic Reticulum Ca2+ Transporting 2 | 488 | One of the SERCA Ca(2+)-ATPases, which are intracellular pumps located in the sarcoplasmic or endoplasmic reticula of muscle cells. Catalyzes the hydrolysis of ATP which is coupled with the translocation of calcium from the cytosol into the sarcoplasmic reticulum lumen, and is involved in regulation of the contraction/relaxation cycle of muscle cells. | ([2](#_ENREF_2),[4](#_ENREF_4),[5](#_ENREF_5)) |
| BUB3 | BUB3, Mitotic Checkpoint Protein | 9184 | A member of the mitotic checkpoint complex (MCC), which is the central activator of the mitotic spindle assembly checkpoint (SAC). The SAC controls metaphase to anaphase transition by guaranteeing the fidelity of chromosome segregation. | ([2](#_ENREF_2),[5-7](#_ENREF_5)) |
| DNTTIP1 | Deoxynucleotidyltransferase Terminal Interacting Protein 1 | 116092 | Binds DNA and enhances the activity of terminal deoxynucleotidyltransferase (DNTT), which is a DNA polymerase that catalyzes the polymerization of DNA in the absence of a DNA template. Also acts as a transcriptional regulator and may recruit Histone Deacetylase 1 (HDAC1) complexes to nucleosomes or naked DNA. | ([2](#_ENREF_2),[5](#_ENREF_5)) |
| DSP | Desmoplakin | 1832 | Involved in the organization of desmosomes, which are protein junctional complexes specialized for cell-to-cell adhesion. | ([2](#_ENREF_2),[5](#_ENREF_5),[8](#_ENREF_8),[9](#_ENREF_9)) |
| HNRNPC | Heterogeneous Nuclear Ribonucleoprotein C (C1/C2) | 3183 | The founding member of the hnRNPs family - the most abundant and promiscuous RBPs. hnRNPs share important roles in multiple aspects of nucleic acid metabolism, including alternative splicing, mRNA stability and transport, and translational regulation. In the DDR context, hNRNPc is recruited to DNA damage sites as part of the BRCA1/BRCA2/PALB2 nucleoprotein complex and is required for correct pre-mRNA splicing and elevated expression of several HRR factors. | ([1](#_ENREF_1),[2](#_ENREF_2),[5-7](#_ENREF_5),[9-15](#_ENREF_9)) |
| LRPPRC | Leucine Rich Pentatricopeptide Repeat Containing | 10128 | Functions in different aspects of RNA metabolism, especially in transcriptional regulation of both nuclear and mitochondrial genes. | ([2](#_ENREF_2),[3](#_ENREF_3),[16](#_ENREF_16)) |
| MDC1 | Mediator Of DNA Damage Checkpoint 1 | 9656 | A scaffold protein that regulates the recruitment of other DDR proteins to sites of damage by participating in multiple protein-protein interactions. MDC1 deficiency leads to hypersensitivity of cells to DSB inducing agents, improper activation of G2/M and the intra-S-phase cell-cycle checkpoints, aberrant activation of DNA damage induced apoptosis and inefficient phosphorylation of several DDR regulators. | ([2](#_ENREF_2),[3](#_ENREF_3),[5](#_ENREF_5),[8-11](#_ENREF_8),[17-19](#_ENREF_17)) |
| MPRIP | Myosin Phosphatase Rho Interacting Protein | 23164 | Required for actin cytoskeleton regulation. Targets myosin phosphatase to the actin cytoskeleton. | ([8](#_ENREF_8)) |
| NUP93 | Nucleoporin 93 | 9688 | Members of the nuclear pore complexes (NPCs). NPCs are transport channels that mediate the bidirectional exchange of macromolecules between the nucleus and cytoplasm, and play a role in posttranscriptional regulation, by controlling selective translocation of nuclear mRNA to the cytoplasmic translational machinery. | ([2](#_ENREF_2),[5](#_ENREF_5),[8](#_ENREF_8),[18](#_ENREF_18)) |
| NUP98 | Nucleoporin 98 | 4928 |  | ([2](#_ENREF_2),[8](#_ENREF_8)) |
| PLOD1 | Procollagen-Lysine,2-Oxoglutarate 5-Dioxygenase 1 | 5351 | Catalyze the hydroxylation of lysyl residues in collagen-like peptides. The resultant hydroxylysyl groups are attachment sites for carbohydrates in collagen and thus are critical for the stability of intermolecular crosslinks. |  |
| PLOD2 | Procollagen-Lysine,2-Oxoglutarate 5-Dioxygenase 2 | 5352 |  | ([6](#_ENREF_6),[9](#_ENREF_9)) |
| PRKDC | DNA-PK Catalytic Subunit | 5591 | The catalytic subunit of the DNA PK holoenzyme, which also contains the KU70–KU80 heterodimer. It is best known for its central role in the non-homologous end-joining (NHEJ) DSB repair pathway but is involved also in other processes, such as cell proliferation and regulation of oxidative stress. | ([2](#_ENREF_2),[5](#_ENREF_5),[8-12](#_ENREF_8),[18](#_ENREF_18),[20](#_ENREF_20)) |
| RAB34 | RAB34, Member RAS Oncogene Family | 83871 | Member of the RAB family of proteins, which are small GTPases involved in protein transport. Plays a role in fusion of phagosomes with lysosomes and in phagosome maturation. | ([2](#_ENREF_2),[5](#_ENREF_5),[8](#_ENREF_8),[21](#_ENREF_21)) |
| RAI14 | Retinoic Acid Induced 14 | 26064 | An actin-binding protein and a member of the retinoic acid induced (RAI) protein family. Serves as an adaptor and scaffold protein, associated with cortical actin cytoskeleton, F-actin stress fibers and cell-cell adhesion sites. | ([2](#_ENREF_2),[8](#_ENREF_8),[13](#_ENREF_13),[18](#_ENREF_18)) |
| ATAD3A | ATPase Family, AAA Domain Containing 3A | 55210 | A mitochondrial membrane protein that contributes to mitochondrial metabolism and to the stabilization of large mitochondrial DNA (mtDNA)-protein complexes called nucleoids. |  |
| SNRPF | Small Nuclear Ribonucleoprotein Polypeptide F | 6636 | a core component of the spliceosome that regulates pre-mRNA splicing, | ([2](#_ENREF_2),[5](#_ENREF_5)) |

(b)

| **Gene name** | **Description** | **Gene id** | **Protein function** | **Hits in previous screens for DDR players (refs.)** |
| --- | --- | --- | --- | --- |
| PABPC1 | Poly(A) Binding Protein Cytoplasmic 1 | 26986 | The protein shuttles between the nucleus and cytoplasm and binds to the 3' poly(A) tail of eukaryotic messenger RNAs via RNA-recognition motifs. The binding of this protein to poly(A) promotes ribosome recruitment and translation initiation; it is also required for poly(A) shortening which is the first step in mRNA decay. | ([1](#_ENREF_1),[2](#_ENREF_2),[7](#_ENREF_7),[9](#_ENREF_9),[22](#_ENREF_22),[23](#_ENREF_23)) |
| PABPC4 | Poly(A) Binding Protein Cytoplasmic 4 | 8761 | The least studied Poly(A)-binding protein. Unlike other RBPs, that are expressed in most cell types, PABPC4 expression is dependent on the status of cells, such as during T-cell activation and thrombin-activated platelets. Among its related pathways are [mRNA surveillance](http://pathcards.genecards.org/card/mrna_surveillance_pathway) and [RNA transport](http://pathcards.genecards.org/card/rna_transport). It also regulates telomerase activity and cell growth in certain cancer cell lines. | ([2](#_ENREF_2),[21](#_ENREF_21),[22](#_ENREF_22)) |
| SKIV2L2 | Ski2 Like RNA Helicase 2 | 23517 | The RNA helicase subunit of the nuclear exosome targeting (NEXT) complex, which also includes RBM7 and ZCCHC8. . The NEXT complex is a key cofactor of the RNA-exosome complex which is an RNA processing and degradation apparatus. | ([2](#_ENREF_2),[9](#_ENREF_9)) |
| ZC3H11A | Zinc Finger CCCH-Type Containing 11A | 9877 | ZC3H11A’s function is not well characterized. Among its related pathways are mRNA [transport](http://pathcards.genecards.org/card/transport_of_mature_transcript_to_cytoplasm) and  [transcription termination](http://pathcards.genecards.org/card/rna_polymerase_ii_transcription_termination). | ([2](#_ENREF_2),[8](#_ENREF_8),[9](#_ENREF_9),[11](#_ENREF_11),[24](#_ENREF_24)) |
| ZC3H14 | Zinc Finger CCCH-Type Containing 14 | 79882 | A poly(A)-binding protein that regulates poly(A) tail length and the expression of specific target mRNAs. May influence mRNA stability, nuclear export, and translation. | ([2](#_ENREF_2),[9](#_ENREF_9),[11](#_ENREF_11),[13](#_ENREF_13),[18](#_ENREF_18)) |
| ZC3H3 | Zinc Finger CCCH-Type Containing 3 | 23144 | Regulates mRNA polyadenylation and is required for mRNA export from the nucleus. | ([8](#_ENREF_8)) |
| ZCCHC8 | Zinc Finger CCHC-Type Containing 8 | 55596 | The scaffolding subunit of the nuclear exosome targeting (NEXT) complex, which also includes RBM7 and SKIV2L2. The NEXT complex is a key cofactor of the RNA-exosome complex which is an RNA processing and degradation apparatus. | ([5](#_ENREF_5),[9](#_ENREF_9)) |

(c)

| **Gene name** | **Gene id** | **Group (IP:Ab/treatment)** |
| --- | --- | --- |
| BUD31 | 8896 | UT, total-PAB Ab |
| NOA1 | 84273 | UT, total-PAB Ab |
| OXA1L | 5018 | UT, total-PAB Ab |
| PLRG1 | 5356 | UT, total-PAB Ab |
| RBM22 | 55696 | UT, total-PAB Ab |
| TOE1 | 114034 | UT, total-PAB Ab |
| B4DLR3 |  | UT, total-PAB Ab |
| U2SURP | 23350 | UT, total-PAB Ab |
| ATAD3B | 83858 | NCS, total-PAB Ab |
| AURKAIP1 | 54998 | NCS, total-PAB Ab |
| DKC1 | 1736 | NCS, total-PAB Ab |
| ETV6 | 2120 | NCS, total-PAB Ab |
| GADD45GIP1 | 90480 | NCS, total-PAB Ab |
| GAR1 | 54433 | NCS, total-PAB Ab |
| HIST1H2BB | 3018 | NCS, total-PAB Ab |
| HNRNPR | 10236 | NCS, total-PAB Ab |
| HNRNPU | 3192 | NCS, total-PAB Ab |
| ICT1 | 3396 | NCS, total-PAB Ab |
| MORF4L1 | 10933 | NCS, total-PAB Ab |
| SYNCRIP | 10492 | NCS, total-PAB Ab |
| SLIRP | 81892 | NCS, total-PAB Ab |
| SMC1A | 8243 | NCS, total-PAB Ab |
| MVP | 9961 | UT/NCS, total-PAB Ab |
| PTCD3 | 55037 | UT/NCS, total-PAB Ab |
| SMC3 | 9126 | UT/NCS, total-PAB Ab |
| SMCR7L | 54471 | UT/NCS, total-PAB Ab |
| YBX1 | 4904 | UT/NCS, total-PAB Ab |
| YBX3 | 8531 | UT/NCS, total-PAB Ab |

**Table S3.** List of PABPN1 protein-protein interactors in UT or NCS-treated cells, using total-PABPN1 or phospho-PABPN1 antibodies. The list is divided to subgroups according to the treatment and antibody that was used. (a) Proteins that precipitated with pPABPN1 after NCS treatment. (b) Proteins that precipitated with pPABPN1 after NCS treatment cells and also with total PABPN1 in untreated and in NCS-treated cells. (c) Proteins that precipitated with total PABPN1 in untreated or in NCS-treated cells. The background colors refer to the subgroup defined in the 3^rd^ column.

**References**

1. Adamson, B., Smogorzewska, A., Sigoillot, F.D., King, R.W. and Elledge, S.J. (2012) A genome-wide homologous recombination screen identifies the RNA-binding protein RBMX as a component of the DNA-damage response. *Nature cell biology*, **14**, 318-328.

2. Elia, A.E., Boardman, A.P., Wang, D.C., Huttlin, E.L., Everley, R.A., Dephoure, N., Zhou, C., Koren, I., Gygi, S.P. and Elledge, S.J. (2015) Quantitative Proteomic Atlas of Ubiquitination and Acetylation in the DNA Damage Response. *Molecular cell*, **59**, 867-881.

3. Kolas, N.K., Chapman, J.R., Nakada, S., Ylanko, J., Chahwan, R., Sweeney, F.D., Panier, S., Mendez, M., Wildenhain, J., Thomson, T.M. *et al.* (2007) Orchestration of the DNA-damage response by the RNF8 ubiquitin ligase. *Science*, **318**, 1637-1640.

4. Emanuele, M.J., Elia, A.E., Xu, Q., Thoma, C.R., Izhar, L., Leng, Y., Guo, A., Chen, Y.N., Rush, J., Hsu, P.W. *et al.* (2011) Global identification of modular cullin-RING ligase substrates. *Cell*, **147**, 459-474.

5. Boeing, S., Williamson, L., Encheva, V., Gori, I., Saunders, R.E., Instrell, R., Aygun, O., Rodriguez-Martinez, M., Weems, J.C., Kelly, G.P. *et al.* (2016) Multiomic Analysis of the UV-Induced DNA Damage Response. *Cell reports*.

6. Paulsen, R.D., Soni, D.V., Wollman, R., Hahn, A.T., Yee, M.C., Guan, A., Hesley, J.A., Miller, S.C., Cromwell, E.F., Solow-Cordero, D.E. *et al.* (2009) A genome-wide siRNA screen reveals diverse cellular processes and pathways that mediate genome stability. *Molecular cell*, **35**, 228-239.

7. Stirling, P.C., Bloom, M.S., Solanki-Patil, T., Smith, S., Sipahimalani, P., Li, Z., Kofoed, M., Ben-Aroya, S., Myung, K. and Hieter, P. (2011) The complete spectrum of yeast chromosome instability genes identifies candidate CIN cancer genes and functional roles for ASTRA complex components. *PLoS genetics*, **7**, e1002057.

8. Beli, P., Lukashchuk, N., Wagner, S.A., Weinert, B.T., Olsen, J.V., Baskcomb, L., Mann, M., Jackson, S.P. and Choudhary, C. (2012) Proteomic investigations reveal a role for RNA processing factor THRAP3 in the DNA damage response. *Molecular cell*, **46**, 212-225.

9. Matsuoka, S., Ballif, B.A., Smogorzewska, A., McDonald, E.R., 3rd, Hurov, K.E., Luo, J., Bakalarski, C.E., Zhao, Z., Solimini, N., Lerenthal, Y. *et al.* (2007) ATM and ATR substrate analysis reveals extensive protein networks responsive to DNA damage. *Science*, **316**, 1160-1166.

10. Bennetzen, M.V., Larsen, D.H., Bunkenborg, J., Bartek, J., Lukas, J. and Andersen, J.S. (2010) Site-specific phosphorylation dynamics of the nuclear proteome during the DNA damage response. *Molecular & cellular proteomics : MCP*, **9**, 1314-1323.

11. Bensimon, A., Schmidt, A., Ziv, Y., Elkon, R., Wang, S.Y., Chen, D.J., Aebersold, R. and Shiloh, Y. (2010) ATM-dependent and -independent dynamics of the nuclear phosphoproteome after DNA damage. *Science signaling*, **3**, rs3.

12. Hurov, K.E., Cotta-Ramusino, C. and Elledge, S.J. (2010) A genetic screen identifies the Triple T complex required for DNA damage signaling and ATM and ATR stability. *Genes & development*, **24**, 1939-1950.

13. Jungmichel, S., Rosenthal, F., Altmeyer, M., Lukas, J., Hottiger, M.O. and Nielsen, M.L. (2013) Proteome-wide identification of poly(ADP-Ribosyl)ation targets in different genotoxic stress responses. *Molecular cell*, **52**, 272-285.

14. Lovejoy, C.A., Xu, X., Bansbach, C.E., Glick, G.G., Zhao, R., Ye, F., Sirbu, B.M., Titus, L.C., Shyr, Y. and Cortez, D. (2009) Functional genomic screens identify CINP as a genome maintenance protein. *Proceedings of the National Academy of Sciences of the United States of America*, **106**, 19304-19309.

15. Kavanaugh, G., Ye, F., Mohni, K.N., Luzwick, J.W., Glick, G. and Cortez, D. (2015) A whole genome RNAi screen identifies replication stress response genes. *DNA repair*, **35**, 55-62.

16. Piwko, W., Olma, M.H., Held, M., Bianco, J.N., Pedrioli, P.G., Hofmann, K., Pasero, P., Gerlich, D.W. and Peter, M. (2010) RNAi-based screening identifies the Mms22L-Nfkbil2 complex as a novel regulator of DNA replication in human cells. *The EMBO journal*, **29**, 4210-4222.

17. Chou, D.M., Adamson, B., Dephoure, N.E., Tan, X., Nottke, A.C., Hurov, K.E., Gygi, S.P., Colaiacovo, M.P. and Elledge, S.J. (2010) A chromatin localization screen reveals poly (ADP ribose)-regulated recruitment of the repressive polycomb and NuRD complexes to sites of DNA damage. *Proceedings of the National Academy of Sciences of the United States of America*, **107**, 18475-18480.

18. Stokes, M.P., Rush, J., Macneill, J., Ren, J.M., Sprott, K., Nardone, J., Yang, V., Beausoleil, S.A., Gygi, S.P., Livingstone, M. *et al.* (2007) Profiling of UV-induced ATM/ATR signaling pathways. *Proceedings of the National Academy of Sciences of the United States of America*, **104**, 19855-19860.

19. Sirbu, B.M., McDonald, W.H., Dungrawala, H., Badu-Nkansah, A., Kavanaugh, G.M., Chen, Y., Tabb, D.L. and Cortez, D. (2013) Identification of proteins at active, stalled, and collapsed replication forks using isolation of proteins on nascent DNA (iPOND) coupled with mass spectrometry. *The Journal of biological chemistry*, **288**, 31458-31467.

20. Smogorzewska, A., Desetty, R., Saito, T.T., Schlabach, M., Lach, F.P., Sowa, M.E., Clark, A.B., Kunkel, T.A., Harper, J.W., Colaiacovo, M.P. *et al.* (2010) A genetic screen identifies FAN1, a Fanconi anemia-associated nuclease necessary for DNA interstrand crosslink repair. *Molecular cell*, **39**, 36-47.

21. Cotta-Ramusino, C., McDonald, E.R., 3rd, Hurov, K., Sowa, M.E., Harper, J.W. and Elledge, S.J. (2011) A DNA damage response screen identifies RHINO, a 9-1-1 and TopBP1 interacting protein required for ATR signaling. *Science*, **332**, 1313-1317.

22. Boucas, J., Fritz, C., Schmitt, A., Riabinska, A., Thelen, L., Peifer, M., Leeser, U., Nuernberg, P., Altmueller, J., Gaestel, M. *et al.* (2015) Label-Free Protein-RNA Interactome Analysis Identifies Khsrp Signaling Downstream of the p38/Mk2 Kinase Complex as a Critical Modulator of Cell Cycle Progression. *PloS one*, **10**, e0125745.

23. Moudry, P., Watanabe, K., Wolanin, K.M., Bartkova, J., Wassing, I.E., Watanabe, S., Strauss, R., Troelsgaard Pedersen, R., Oestergaard, V.H., Lisby, M. *et al.* (2016) TOPBP1 regulates RAD51 phosphorylation and chromatin loading and determines PARP inhibitor sensitivity. *The Journal of cell biology*, **212**, 281-288.

24. Izhar, L., Adamson, B., Ciccia, A., Lewis, J., Pontano-Vaites, L., Leng, Y., Liang, A.C., Westbrook, T.F., Harper, J.W. and Elledge, S.J. (2015) A Systematic Analysis of Factors Localized to Damaged Chromatin Reveals PARP-Dependent Recruitment of Transcription Factors. *Cell reports*, **11**, 1486-1500.
